# Blockage of Akt activation suppresses cadmium-induced renal tubular cellular damages through aggrephagy in HK-2 cells

**DOI:** 10.1038/s41598-024-64579-3

**Published:** 2024-06-24

**Authors:** Kota Fujiki, K. Tanabe, S. Suzuki, A. Mochizuki, M. Mochizuki-Kashio, T. Sugaya, T. Mizoguchi, M. Itoh, A. Nakamura-Ishizu, H. Inamura, M. Matsuoka

**Affiliations:** 1 Department of Hygiene and Public Health, Tokyo Women’s Medical University, 8-1 Kawada-cho, Shinjuku-ku, Tokyo, 162-8666 Japan; 2 Institute for Comprehensive Medical Sciences, Tokyo Women’s Medical University, Tokyo, 162-8666 Japan; 3 Graduate School of Pharmaceutical Sciences, Chiba University, Chiba, 260-8675 Japan; 4 Department of Bio-Medical Engineering, School of Engineering, Tokai University, Kanagawa, 259-1143 Japan; 5 Department of Microanatomy and Development Biology, Tokyo Women’s Medical University, Tokyo, 162-8666 Japan; 6 Division of Nephrology and Hypertension, St. Marianna University School of Medicine, Kanagawa, 216-8511 Japan

**Keywords:** Cadmium, Akt, Cell death, Renal proximal tubular cells, Aggresome, Cell death, Cell signalling, Mechanisms of disease, Toxin-induced nephropathy

## Abstract

We have reported that an environmental pollutant, cadmium, promotes cell death in the human renal tubular cells (RTCs) through hyperactivation of a serine/threonine kinase Akt. However, the molecular mechanisms downstream of Akt in this process have not been elucidated. Cadmium has a potential to accumulate misfolded proteins, and proteotoxicity is involved in cadmium toxicity. To clear the roles of Akt in cadmium exposure-induced RTCs death, we investigated the possibility that Akt could regulate proteotoxicity through autophagy in cadmium chloride (CdCl_2_)-exposed HK-2 human renal proximal tubular cells. CdCl_2_ exposure promoted the accumulation of misfolded or damaged proteins, the formation of aggresomes (pericentriolar cytoplasmic inclusions), and aggrephagy (selective autophagy to degrade aggresome). Pharmacological inhibition of Akt using MK2206 or Akti-1/2 enhanced aggrephagy by promoting dephosphorylation and nuclear translocation of transcription factor EB (TFEB)/transcription factor E3 (TFE3), lysosomal transcription factors. TFEB or TFE3 knockdown by siRNAs attenuated the protective effects of MK2206 against cadmium toxicity. These results suggested that aberrant activation of Akt attenuates aggrephagy via TFEB or TFE3 to facilitate CdCl_2_-induced cell death. Furthermore, these roles of Akt/TFEB/TFE3 were conserved in CdCl_2_-exposed primary human RTCs. The present study shows the molecular mechanisms underlying Akt activation that promotes cadmium-induced RTCs death.

## Introduction

Preventing renal tubular cells (RTCs) death due to environmental stress or nephrotoxic substances is a potential therapeutic strategy for treating nephropathy. Cadmium is an occupational and environmental pollutant that can damage various organs, especially causing renal dysfunction accompanied by renal proximal tubular cell death^1–8^. The major sources of chronic cadmium exposure in humans are rechargeable nickel–cadmium batteries, smoking, drinking water, and food^9–11^. Orally ingested cadmium accumulates in the body, especially in the kidneys^12^, and its overaccumulation in kidney damages the proximal tubules and induces RTCs death^3,5,13^. Cadmium exposure reportedly disturbs various intracellular signaling to induce RTCs death. Previously, we have identified activin receptor-like kinase (ALK) 4 or 5, a receptor for activins or transforming growth factor (TGF) β, and Notch1, a transcription factor and receptor for Notch ligands such as Jagged1, as key molecules in cadmium toxicity^14,15^. Briefly, in cadmium-exposed cells, ALK4/5 phosphorylates a transcription factor Smad3 and activates a serine/threonine kinase Akt (also known as protein kinase B) to promote cell death. In addition, cadmium exposure accumulates Notch1-intracellular domain (ICD) by the cleavage of γ-secretase complex in Notch1, and Notch1-ICD subsequently upregulates the expression of a transcription factor Snail and activates Akt to facilitate cell death. However, intracellular events downstream of Akt signaling in cadmium stress response remain elusive.

Failure to refold or degrade excess cellular misfolded proteins by chaperons or the ubiquitin–proteasome system (UPS) results in the formation of aggrsomes^16^. Aggresomes are pericentriolar cytoplasmic inclusions containing misfolded and ubiquitinated proteins surrounded by intermediate filaments such as Vimentin. Aggresomes are formed by retrograde transport of the misfolded protein microaggregates from the cell periphery towards the microtubule organizing center (MTOC) via microtubules^17,18^ and degraded by aggrephagy, a selective autophagic clearance process^19^. Aggrephagy seems to be a cytoprotective system to cope with the accumulation of misfolded protein aggregates when chaperones and UPS are impaired^20^. Protein aggregation and aggresome formation are observed in toxicant-exposed and virus-infected cells^21–24^.

Clearance of aggresomes relies on lysosome biogenesis and autophagy. The microphthalmia family of transcription factors (MiT/TFEs), including transcription factor EB (TFEB), transcription factor E3 (TFE3), and microphthalmia-associated transcription factor (MITF), are major regulators of lysosome biogenesis and autophagy. MiT/TFEs induce lysosomal/autophagic target gene transcriptions by binding at CLEAR (Coordinated Lysosomal Expression And Regulation) motifs. Phosphorylation of MiT/TFEs at several residues by Akt and its substrate, mechanistic target of rapamycin complex (mTORC) 1, promotes MiT/TFEs cytoplasmic retention leading to decrease in lysosome biogenesis and autophagy^25–31^. Previous reports have shown that some internal and external stresses such as nutrient starvation, endoplasmic reticulum stress, and lysosomal membrane damage activate MiT/TFEs in an Akt/mTORC1-dependent or -independent manner^29,32–34^, but the roles of MiT/TFEs in cell death varies with the type of stress.

To clarify how Akt signaling mediates cadmium-induced RTCs death, we analyzed cell death of HK-2 human proximal RTCs or primary human renal proximal tubule epithelial cells (RPTECs) exposed to cadmium. Based on the previous knowledge, we postulated that Akt might regulate proteotoxicity through autophagy in cadmium-exposed RTCs, and we tested this possibility in this study. We observed that aggresome is formed in cadmium chloride (CdCl_2_)-exposed HK-2 cells. Pharmacological inhibition of Akt promoted aggrephagy via TFEB/TFE3 and mitigated cadmium-induced proteotoxicity and cell death. We also confirmed the roles of Akt/TFEB/TFE3 in CdCl_2_-induced RPTECs death. While controversial roles of Akt in cadmium toxicity have been reported previously^14,15,29,35,36^, our findings suggest the inhibitory role of hyperactivated Akt for cell survival in cadmium stress response.

## Results

### CdCl_2_ exposure promoted aggresome formation in HK-2 cells

**Figure 1:**
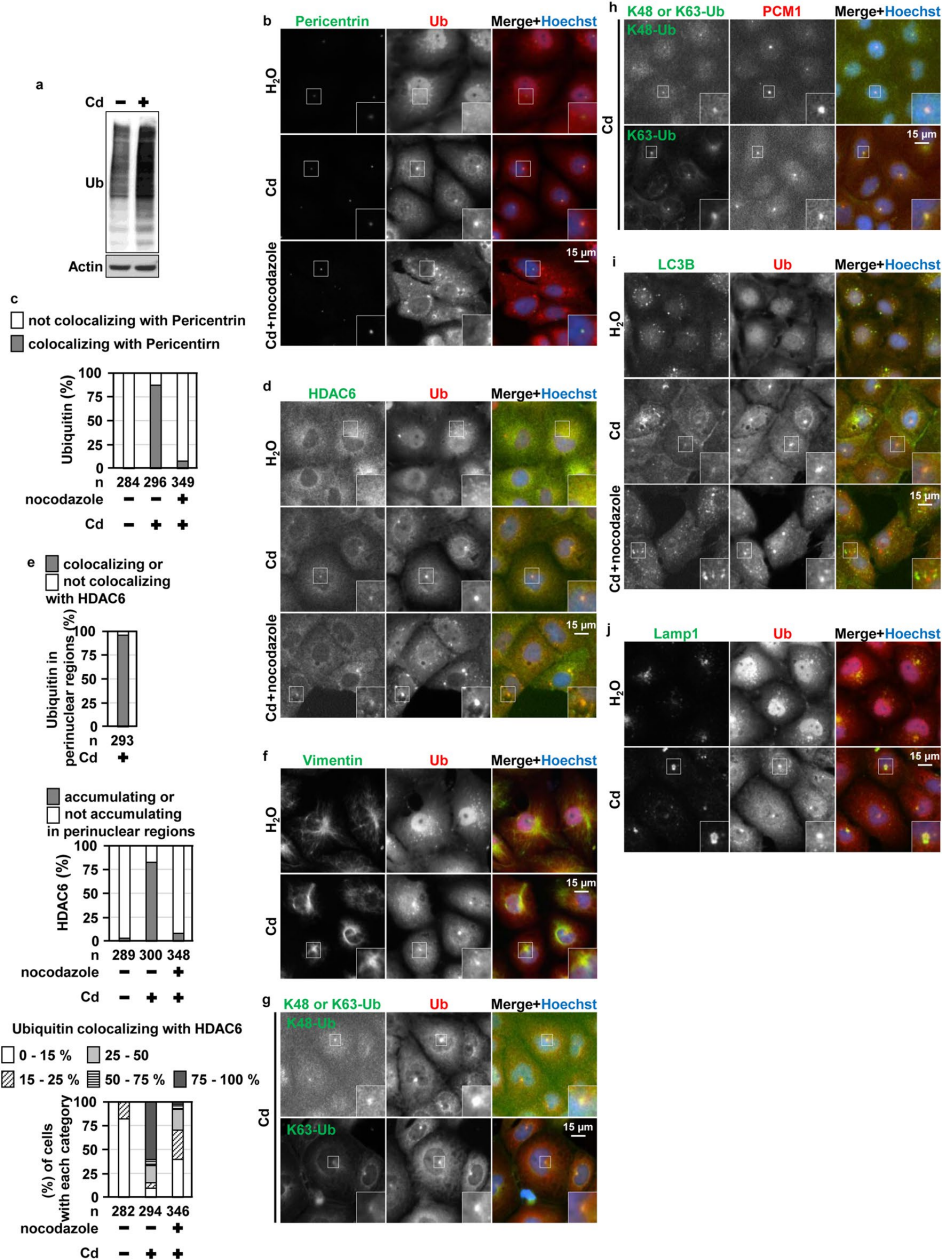
Cadmium exposure promotes aggresome formation and aggrephagy in HK-2 cells. (**a**) Cells were incubated with 25 μM CdCl_2_ (Cd) for 6 h for western blotting. Immunoblots shown are representative of at least three independent experiments. The displayed blots were cropped, and the full-length blots are shown in Supplementary Fig. S1. (**b**–**e**,**i**) Cells were incubated with 0.1% DMSO or 10 μM nocodazole for 0.5 h, and then incubated with or without 25 μM CdCl_2_ for 6 h. Cells were stained with anti-Pericentrin (**b**), HDAC6 (**d**) or anti-LC3B (**i**) (green), anti-Ubiquitin (Ub: red) antibodies, and Hoechst for DNA (blue). Percentages of the cells containing Ubiquitin puncta colocalizing with Pericentrin puncta were quantified (**c**). Percentages of the Ubiquitin puncta colocalizing with HDAC6 in the cells were quantified and divided into the 5 categories. Percentages of the cells in each category are listed in (**d**). (**f**,**j**) Cells were incubated with or without 25 μM CdCl_2_ for 6 h. Cells were stained with anti-Vimentin (**f**) or anti-Lamp1 (green) (**j**), anti-Ubiquitin (Ub: red) antibodies, and Hoechst for DNA (blue). (**g**,**h**) Cells were incubated with 25 μM CdCl_2_ for 6 h. Cells were stained with anti-K48- or K63-Ubiquitin (K48-Ub or K63-Ub: green), anti-Ubiquitin (Ub) or anti-PCM1 (Red) antibodies and Hoechst for DNA (blue). Images are representative of three independent experiments. Scale bars 15 µm. The numbers (n) of cells examined are shown.

Cadmium exposure increases misfolded or damaged proteins and cells with defects in protein quality control, such as defective ubiquitin-conjugating enzymes or proteasome activity, are hypersensitive to cadmium^22–24,37–39^. Akt positively or negatively modulates protein clearance mechanisms^25–31,40–44^. Therefore, we investigated the possible roles of hyperactivated Akt in cadmium-induced proteotoxicity. First, we monitored the dynamics of polyubiquitinated proteins, known as a marker of misfolded or damaged proteins, in CdCl_2_-exposed HK-2 cells. Consistent with previous reports, ubiquitinated proteins increased in CdCl_2_-exposed cells (Fig. 1a). Microscopy analysis revealed that puncta of ubiquitinated proteins accumulated at perinuclear regions and overlapped with a MTOC marker Pericentrin (Fig. 1b,c). Treatment with nocodazole, a microtubule-disrupting drug, inhibited the accumulation of ubiquitinated proteins at MTOC in CdCl_2_-exposed cells. According to these results, CdCl_2_ exposure facilitated aggresome formation in HK-2 cells, which was also confirmed by the staining for histone deacetylase 6 (HDAC6) and Vimentin, two known aggresome markers^45^ (Fig. 1d–f). By the staining for an MTOC marker pericentriolar material 1, proteins modified with K48- or K63-linked polyubiquitin chains, which are involved in UPS, aggresome formation, or autophagy^19,46^, were confirmed to accumulate at aggresome in CdCl_2_-exposed cells (Fig. 1g,h). We also observed that puncta of microtubule-associated protein 1 light chain 3 beta (LC3B), an autophgosome marker, and lysosome-associated membrane protein (LAMP) 1, a lysosome marker, accumulated at aggresome (Fig. 1i,j), demonstrating that aggrephagy might be induced in CdCl_2_-exposed HK-2 cells.

### Blockage of Akt facilitated aggrephagy via TFEB and TFE3 in CdCl_2_-exposed HK-2 cells

**Figure 2:**
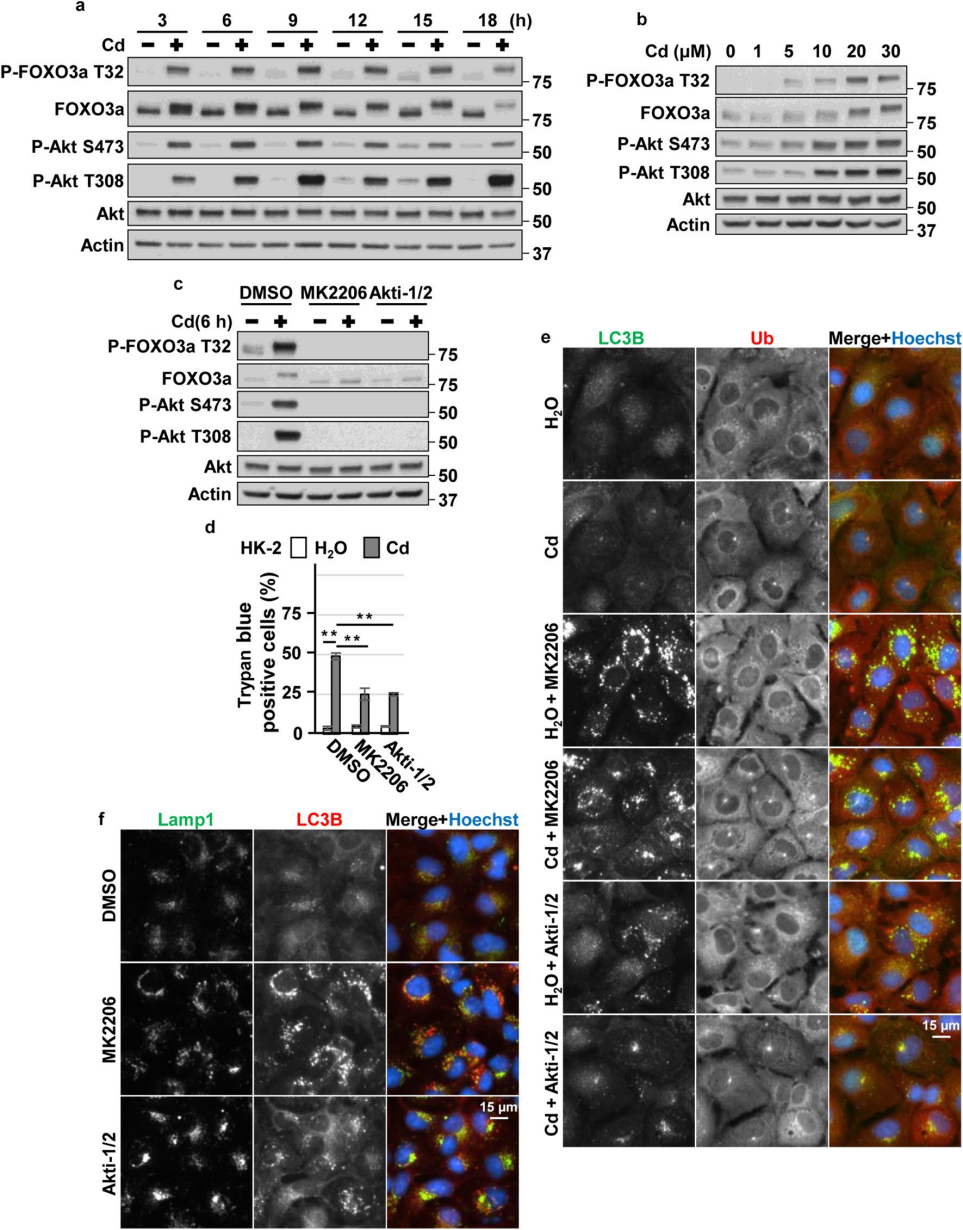
Roles of Akt in cadmium exposure-induced HK-2 cells death. (**a**,**b**) Cells were incubated with 25 μM CdCl_2_ (Cd) for 3–18 h (**a**) or with 1–30 μM CdCl_2_ for 6 h (**b**) for western blotting. (**c**,**d**) Cells were incubated with 0.1% DMSO, 10 μM MK2206, or 10 μM Akti-1/2 for 1 h, and then incubated with or without 25 μM CdCl_2_ for 6 h (**c**) or 27 h (**d**). Cell lysates were subjected to western blotting (**c**). The viability of cells was determined by trypan blue exclusion assay (**d**). (**e**) Cells were incubated with 0.1% DMSO, 10 μM MK2206, or 10 μM Akti-1/2 for 1 h, and then incubated with or without 25 μM CdCl_2_ for 6 h. Cells were stained with anti-LC3B (green), anti-Ubiquitin (Ub: red) antibodies, and Hoechst for DNA (blue). (**f**) Cells were incubated with 0.1% DMSO, 10 μM MK2206, or 10 μM Akti-1/2 for 6 h. Cells were stained with anti-Lamp1 (green), anti-LC3B (Red) antibodies and Hoechst for DNA (blue). Images are representative of three independent experiments. Scale bars: 15 µm. Immunoblots shown are representative of at least three independent experiments. The displayed blots were cropped, and the full-length blots are shown in Supplementary Fig. S1. Each value of trypan blue exclusion assay is the percentage of trypan blue-positive cells and reflects the mean ± SD of at least three experiments with duplicate assays in each experiment. **P < 0.01, significant difference between the samples.

**Figure 3:**
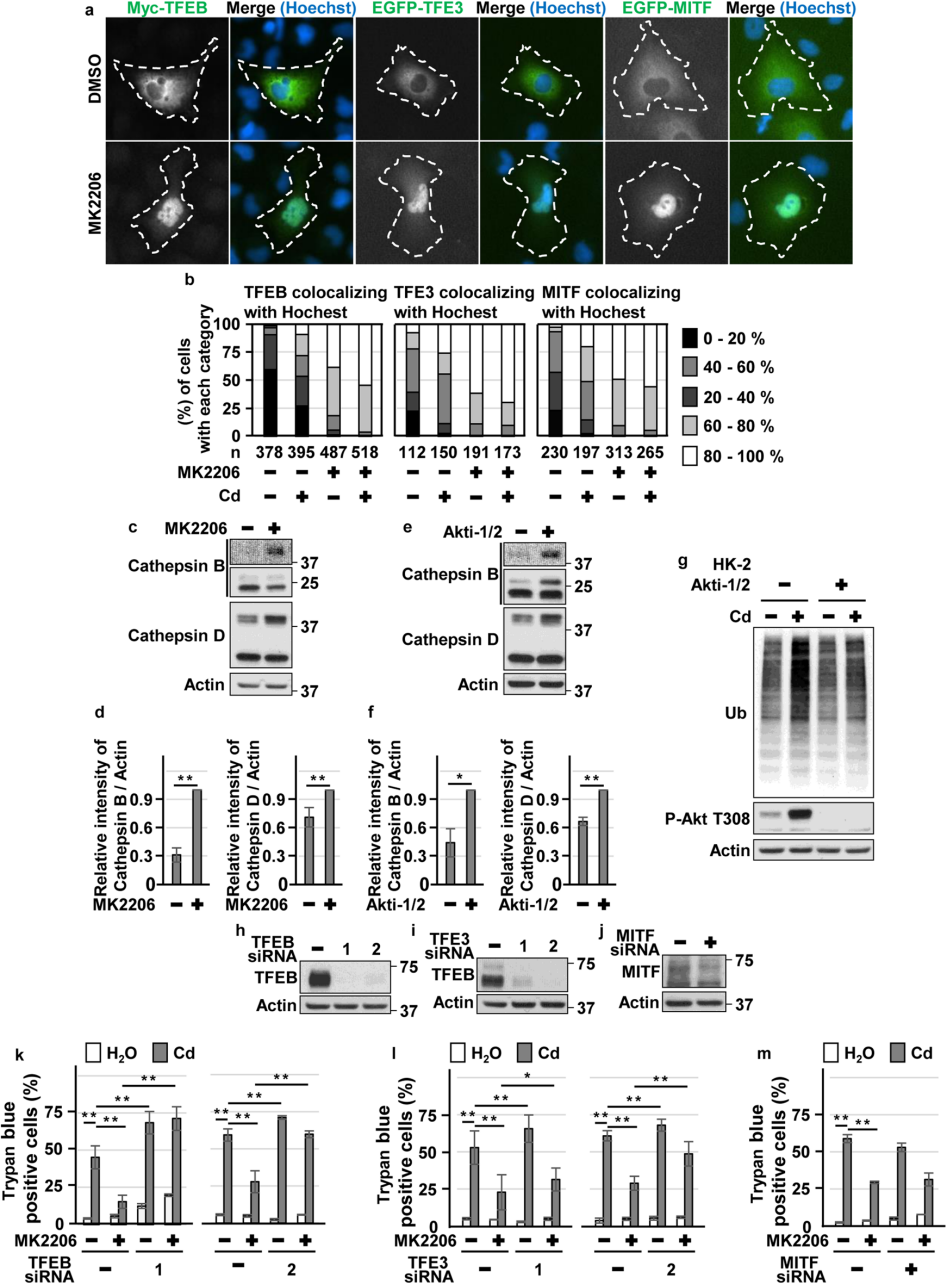
Roles of Akt, TFEB and TFE3 in cadmium exposure-induced HK-2 cells death. (**a**,**b**) Cells transfected with Myc-TFEB, EGFP-TFE3 or EGFP-MITF were incubated with 0.1% DMSO or 10 μM MK2206 for 1 h, and then incubated with (**b**) or without (**a**,**b**) 25 μM CdCl_2_ (Cd) for 2 h. Myc-TFEB-expressing cells were stained with anti-Myc (green) antibodies and Hoechst for DNA (blue). EGFP-TFE3 or EGFP-MITF-expressing cells were stained with Hoechst for DNA (blue). Percentages of the Myc-TFEB, EGFP-TFE3 or EGFP-MITF colocalizing with Hocechst in the cells are quantified and divided into the 5 categories. Percentages of the cells in each category are listed in (**b**). The numbers (n) of cells examined are shown. White dot line indicate cells expressing Myc-TFEB, EGFP-TFE3, or EGFP-MITF. (**c**–**f**) Cells were incubated with 0.1% DMSO, 10 μM MK2206 (**c**,**d**), or 10 μM Akti-1/2 (**e**,**f**) for 6 h for western blotting (**c**–**f**). Results of densitometric analysis (mean ± SD) of at least three independent experiments. (**g**) Cells were incubated with 0.1% DMSO or 10 μM Akti-1/2 for 1 h, and then incubated with or without 25 μM CdCl_2_ (Cd) for 6 h for western blotting. (**h**–**j**) Cells were transfected with control siRNA, TFEB siRNA-1, TFEB siRNA-2 (**h**), TFE3 siRNA-1, TFE3 siRNA-2 (**i**), or MITF siRNA (**j**) for western blotting. (**k**–**m**) Cells transfected with control siRNA, TFEB siRNA-1, TFEB siRNA-2 (**k**), TFE3 siRNA-1, TFE3 siRNA-2 (**l**), or MITF siRNA (**m**) were incubated with 0.1% DMSO or 10 µM MK2206 for 1 h, and then incubated with or without 25 μM CdCl_2_ (Cd) for 24 h. The viability of cells was determined by trypan blue exclusion assay. Images are representative of three independent experiments. Scale bars: 15 µm. Immunoblots shown are representative of at least three independent experiments. The displayed blots were cropped, and the full-length blots are shown in Supplementary Fig. S1. Each value of trypan blue exclusion assay is the percentage of trypan blue-positive cells and reflects the mean ± SD of at least three experiments with duplicate assays in each experiment. *P < 0.05, **P < 0.01, significant difference between the samples.

Consistent with our previous reports^14,15^, CdCl_2_ exposure induced phosphorylation of Akt (at Thr308 or Ser473 residue), both of which are required for Akt activation, in HK-2 cells (Fig 2a,b). CdCl_2_ exposure also induced phosphorylation of forkhead box O (FOXO) 3a at Thr32, which is phosphorylated by Akt^47^. Disruption of Akt signaling using specific Akt inhibitors, MK2206 or Akti-1/2, suppressed CdCl_2_-induced HK-2 cells death (Fig. 2c,d). These results suggested that CdCl_2_ exposure highly activates Akt signaling to promote HK-2 cells death. Next, we investigated the effects of hyperactivated Akt on aggrephagy in CdCl_2_-exposed HK-2 cells. MK2206 or Akti-1/2 treatment increased the number of LC3B puncta, some of which overlapped with LAMP1 puncta, in cells with or without CdCl_2_ exposure (Fig. 2e,f). CdCl_2_ exposure and MK2206 treatment synergistically promoted nuclear translocation of Myc-tagged TFEB, EGFP-TFE3, and EGFP-MITF (Fig. 3a,b). In addition, MK2206 or Akti-1/2 treatment increased the expressions of lysosomal hydrolases Cathepsin B and D, downstream targets of MiT/TFEs (Fig. 3c–f), and mitigated CdCl_2_-induced accumulation of ubiquitinated proteins (Fig. 3g). Furthermore, TFEB or TFE3 depletion but not MITF depletion using specific siRNAs attenuated the protective effects of MK2206 treatment against cadmium toxicity (Fig. 3h–m). These results suggest that blockage of hyperactivated Akt might facilitate aggrephagy and mitigate CdCl_2_-induced HK-2 cells death via TFEB and TFE3.

### Hyperactivated Akt delayed dephosphorylation of TFEB and TFE3 in CdCl_2_-exposed HK-2 cells

**Figure 4:**
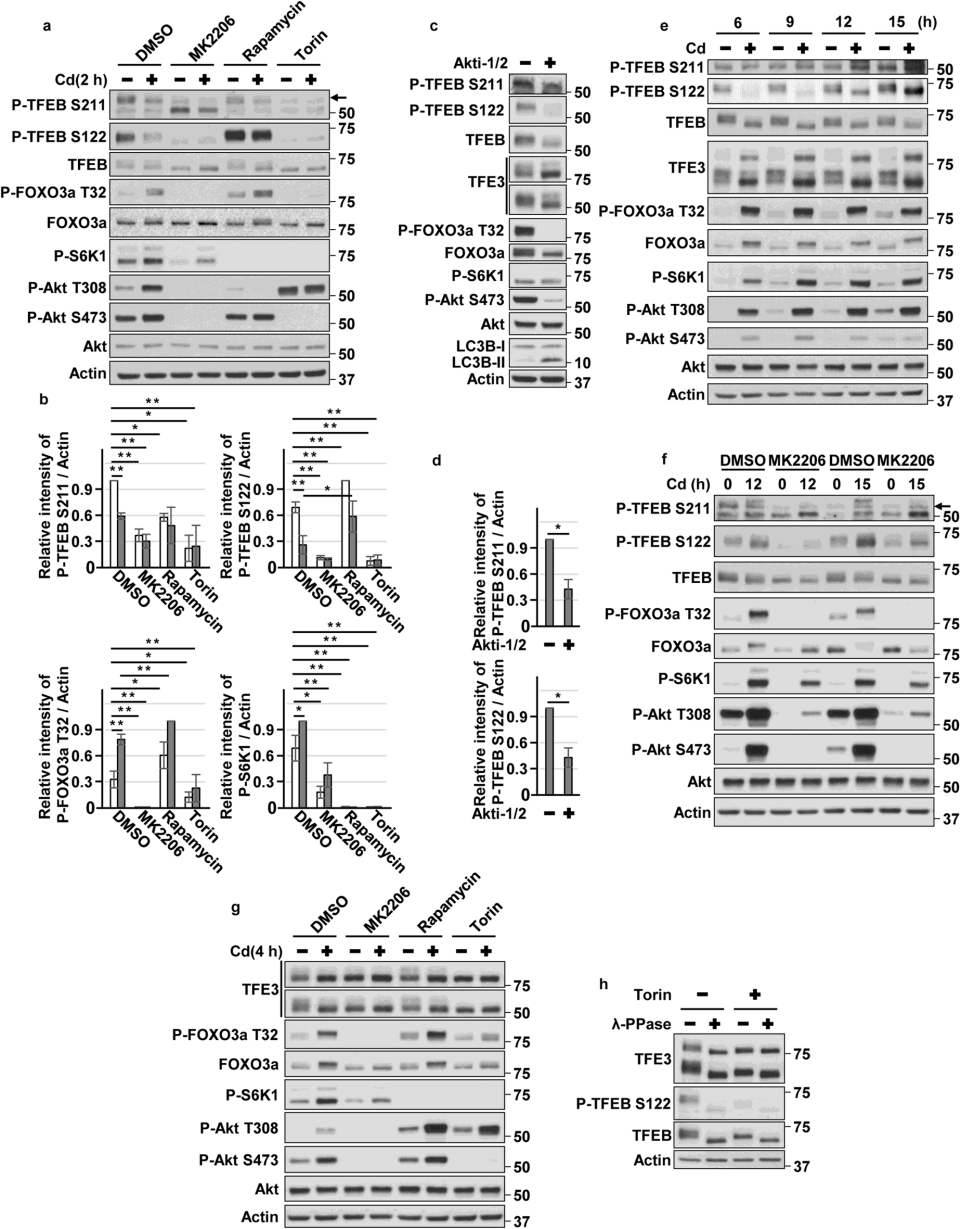
Roles of Akt in TFEB/TFE3 phosphorylation status under cadmium stress. (**a**,**b**,**g**) Cells were incubated with 0.1% DMSO, 10 μM MK2206, 200 ng/ml rapamycin, or 0.5 μM Torin for 1 h, and then incubated with or without 25 μM CdCl_2_ (Cd) for 2 h (**a**,**b**) or 4 h (**g**) for western blotting. (**c**,**d**) HK-2 cells were incubated with 0.1% DMSO or 10 μM Akti-1/2 for 6 h for western blotting. (**e**) HK-2 cells were incubated with or without 25 μM CdCl_2_ (Cd) for the indicated time for western blotting. (**f**) HK-2 cells were incubated with 0.1% DMSO or 10 μM MK2206 for 1 h, and then incubated with or without 25 μM CdCl_2_ (Cd) for 12–15 h for western blotting. (**h**) HK-2 cells were incubated with 0.1% DMSO or 0.5 μM Torin for 4 h, and cells lysates were subsequently processed for phosphatase assay. Cell lysates were subjected to western blotting. Immunoblots shown are representative of at least three independent experiments. The displayed blots were cropped, and the full-length blots are shown in Supplementary Fig. S1. Results of densitometric analysis (mean ± SD) of at least three independent experiments. An arrow indicates the position of P-TFEB S211 (**a**,**f**). *P < 0.05, **P < 0.01, significant difference between the samples.

To clear how hyperactivated Akt regulates TFEB under cadmium stress, we monitored the dynamics of two phosphorylation sites of TFEB at Ser211 and Ser122, which cooperate to inhibit nuclear translocation of TFEB^31^. Consistent with microscopy analysis (Fig. 3a,b), MK2206 or Akti-1/2 treatment decreased the phosphorylation of TFEB at both sites (Fig. 4a–d). Since mTORC1 has been reported to phosphorylate both residues of TFEB^25,31^, we confirmed the effects of Torin, an mTORC1/2 inhibitor, and Rapamycin, an mTORC1 inhibitor, on TFEB phosphorylation. Torin treatment abolished phosphorylation of TFEB-S211 and TFEB-S122 (Fig. 4a,b). But, rapamycin treatment mildly decreased TFEB-S211 phosphorylation and increased TFEB-S122 phosphorylation, even though rapamycin treatment abolished the phosphorylation of an mTORC1 substrate S6 kinase (S6K) 1. These results might be attributed to differences in the pharmacological actions of the allosteric mTORC1 inhibitor rapamycin and the catalytic mTORC1 inhibitor Torin on TFEB^27,30,48^. In contrast, in the context of mTORC1 loss-of-function, MiT/TFEs are highly activated by hyperactivated Akt^49^. In the present study, Akt was highly activated in rapamycin- but not in Torin-treated HK-2 cells, confirmed by phosphorylated FOXO3a levels (Fig. 4a,b). Therefore, these findings combined with the fact that mTORC2 activates Akt through phosphorylation of Akt-S473 raise another possibility that disruption of Akt activity might be necessary to maintain low phosphorylation state of TFEB-S122 in mTORC1-disrupted HK-2 cells. CdCl_2_ exposure transiently dephosphorylated TFEB-S211 and TFEB-S122, and MK2206 treatment delayed the recovery of TFEB phosphorylation at both sites after 15 h of cadmium exposure (Fig. 4e,f). These results suggest that hyperactivated Akt by cadmium exposure might facilitate phosphorylation of TFEB-S211 and TFEB-S122, delay TFEB nuclear translocation, and shorten TFEB nuclear localization time in HK-2 cells. In addition, treatment with MK2206, Akti-1/2, Torin, or CdCl_2_ as well as lambda-phosphatase, a protein serine/threonine/tyrosine phosphatase, increased TFE3 electrophoretic mobility (Fig. 4c,g,h), indicating that inhibition of Akt and mTORC1, or cadmium exposure might facilitate dephosphorylation of TFE3. Based on the results of microscopy analysis (Fig. 3a,b) and TFE3 electrophoretic mobility (Fig. 4c,g,h), hyperactivated Akt by CdCl_2_ exposure might facilitate phosphorylation of TFE3 and delayed nuclear translocation of TFE3.

### Roles of Akt in cadmium exposure-induced RPTECs death

**Figure 5:**
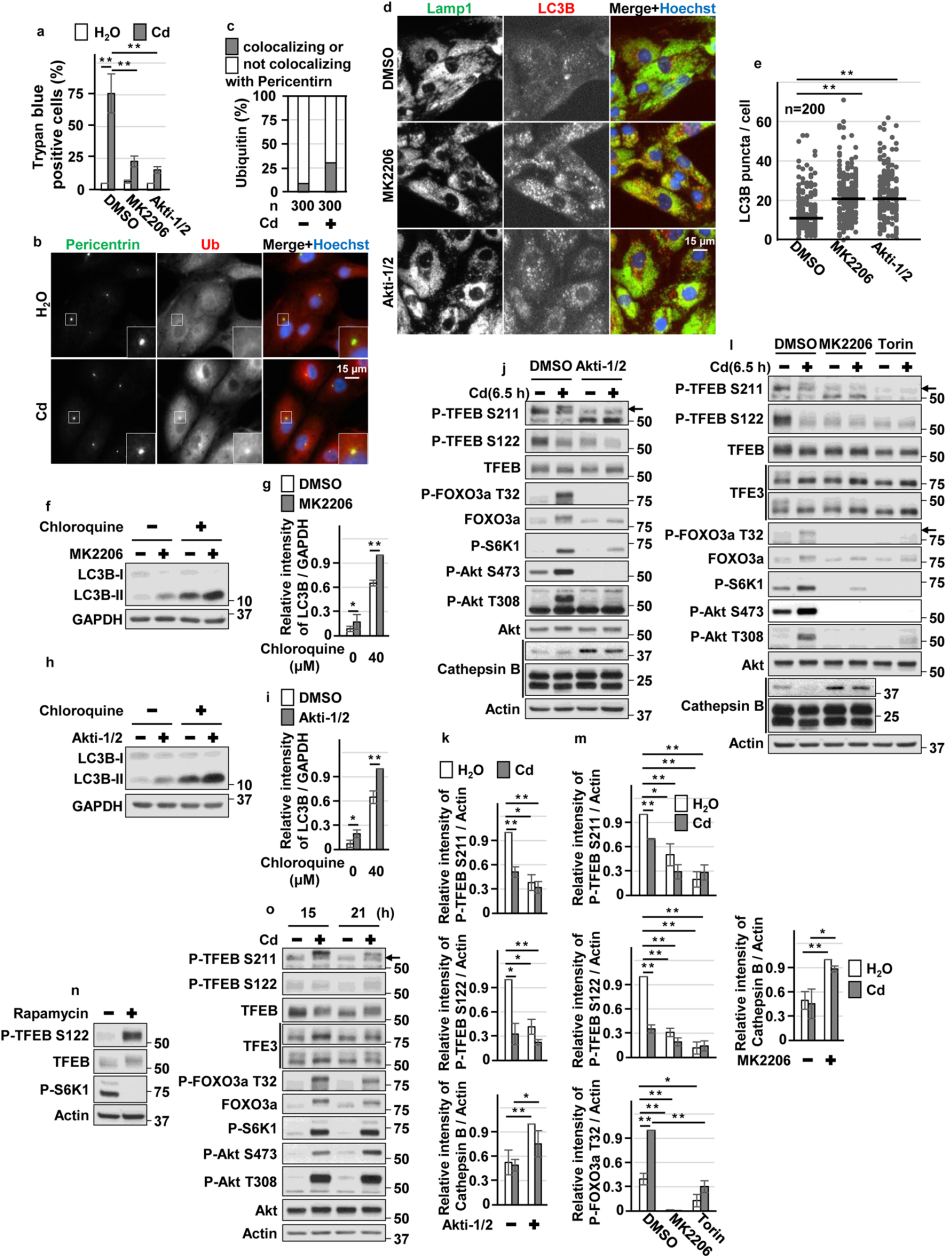
Roles of Akt in cadmium exposure-induced RPTECs death. (**a**) Cells were incubated with 0.1% DMSO, 10 μM MK2206, or 10 μM Akti-1/2 for 1 h, then incubated with or without 25 μM CdCl_2_ (Cd) for 35 h. The viability of cells was determined by trypan blue exclusion assay. Each value of trypan blue exclusion assay is the percentage of trypan blue-positive cells and reflects the mean ± SD of at least three experiments with duplicate assays. (**b**,**c**) Cells were incubated with or without 25 μM CdCl_2_ for 6.5 h. Cells were stained with anti-Pericentrin (green), anti-Ubiquitin (Ub: red) antibodies, and Hoechst for DNA (blue). Percentages of the cells containing Ubiquitin puncta colocalizing with Pericentrin puncta were quantified (**c**). (**d**,**e**) Cells were incubated with 0.1% DMSO, 10 µM MK2206, or 10 µM Akti-1/2 for 6.5 h. Cells were stained with anti-Lamp1 (green), anti-LC3B (Red) antibodies, and Hoechst for DNA (blue) (**d**). The number of LC3B puncta in the cell were counted and listed in (**e**). The data are combined from at least three experiments and compared by the Mann–Whitney U test. The horizontal bars indicate the mean. (**f**–**i**) Cells were incubated with 0.2% DMSO, 10 μM MK2206, 10 µM Akti-1/2 or 40 μM Chloroquine for 4 h for western blotting. (**j–m**) Cells were incubated with 0.1% DMSO, 10 μM Akti-1/2 (**j**,**k**), 10 μM MK2206, or 0.5 μM Torin (**l**,**m**) for 1 h, then incubated with or without 25 μM CdCl_2_ for 6.5 h for western blotting. (**n**) Cells were incubated with 0.1% DMSO or 200 ng/ml rapamycin for 6.5 h for western blotting. (**o**) Cells were incubated with or without 25 μM CdCl_2_ for the indicated time for western blotting. The numbers (n) of cells examined are shown. Scale bars: 15 µm. Immunoblots shown are representative of at least three independent experiments. The displayed blots were cropped, and the full-length blots are shown in Supplementary Fig. S1. Results of densitometric analysis (mean ± SD) of at least three independent experiments. Arrows indicate the positions of P-FOXO3a (**j**) or P-TFEB S211 (**h**,**j**). *P < 0.05, **P < 0.01, significant difference between the samples.

We next confirmed whether Akt regulates aggrephagy in RPTECs, as in HK-2 cells, following exposure to cadmium. MK2206 or Akti-1/2 treatment suppressed CdCl_2_-induced RPTECs death (Fig. 5a). Microscopy analysis revealed ubiquitinated proteins accumulated at MTOC in CdCl_2_-exposed RPTECs (Fig. 5b,c). MK2206 or Akti-1/2 treatment increased the number of LC3B puncta in RPTECs (Fig. 5d,e), and treatment with chloroquine, an autophagy inhibitor, increased the levels of phosphatidylethanolamine-conjugated form of LC3B (LC3B-II), an indicator of autophagosome formation, in RPTECs treated with MK2206 or Akti-1/2 compared with control cells (Fig. 5f–i). These findings indicated high autophagy flux in Akt-inhibited RPTECs. MK2206 or Akti-1/2 treatment increased the expression of Cathepsin B, diminished the phosphorylation of TFEB-S211 and TFEB-S122, and facilitated TFE3 electrophoretic mobility in RPTECs (Fig. 5j–m). Treatment with Torin but not rapamycin reduced the phosphorylation of TFEB-S122 (Fig. 5l–n), and transient decrease in phosphorylation of TFEB-S122 was observed in CdCl_2_-exposed RPTECs (Fig. 5o). These results suggest that Akt might regulate aggrephagy in RPTECs, as in HK-2 cells following exposure to CdCl_2_.

### Effects of a calcineurin inhibitor (FK506) on TFEB/TFE3 phosphorylation status under cadmium stress

**Figure 6:**
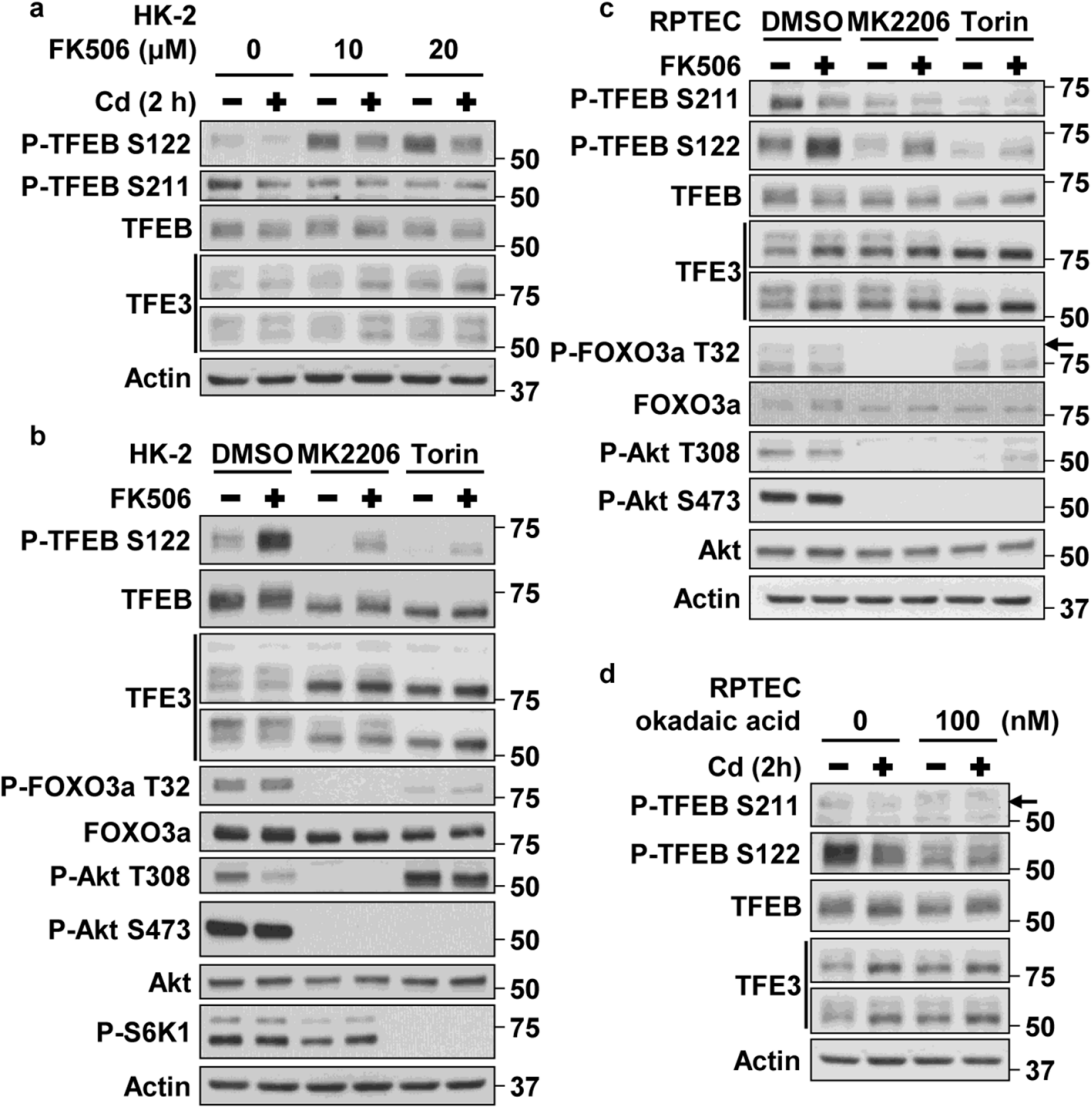
Effects of a calcineurin inhibitor (FK506) on TFEB/TFE3 phosphorylation status under cadmium stress. (**a**) HK-2 cells were incubated with 0.1% DMSO or 10–20 µM FK506 for 1 h, and then incubated with or without 25 µM CdCl_2_ (Cd) for 2 h for western blotting. (**b**,**c**) HK-2 cells (**b**) and RPTECs (**c**) were incubated with 0.1% DMSO, 10 µM MK2206, or 0.5 µM Torin for 4 h and then incubated with or without 20 µM FK506 for 3 h for western blotting. (**d**) RPTECs were incubated with 0.1% DMSO or 100 nM okadaic acid for 1 h, and then incubated with or without 25 µM CdCl_2_ for 2 h for western blotting. Immunoblots shown are representative of at least three independent experiments. The displayed blots were cropped, and the full-length blots are shown in Supplementary Fig. S1. Arrows indicate the positions of P-FOXO3a (**c**) or P-TFEB-S211 (**d**).

Because CdCl_2_ exposure facilitated dephosphorylation of TFEB and TFE3 despite hyperactivation of Akt and mTORC1, we hypothesized that some phosphatases were involved in this regulation. Since calcineurin and protein phosphatase 2 (PP2) dephosphorylate or activate TFEB and TFE3^32,33,50^, we investigated the effects of FK506, a calcineurin inhibitor, or okadaic acid, a PP2 inhibitor, on phosphorylation status of TFEB and TFE3 electrophoretic mobility in CdCl_2_-exposed HK-2 cells. As a result, FK506 treatment increased the phosphorylation of TFEB-S122 in cells with or without CdCl_2_ exposure (Fig. 6a, data not shown). In addition, FK506 partially attenuated the suppressive effects of MK2206 or Torin on the phosphorylation of TFEB-S122 without changing the activity of Akt and mTORC1 (Fig. 6b). Treatment with FK506 but not okadaic acid elevated the phosphorylation of TFEB-S122 (Fig. 6c,d). These results suggest that calcineurin might dephosphorylate TFEB-S122 to activate TFEB in CdCl_2_-exposed HK-2 cells and RPTECs.

## Discussion

In this study, we suggested the molecular mechanisms underlying aberrant activation of Akt that facilitates cadmium-induced RTCs death. Cadmium exposure increased misfolded or damaged proteins and facilitated aggresome formation in RTCs. Blockage of hyperactivated Akt attenuated the phosphorylation of TFEB/TFE3, improved clearance of protein aggregates via aggrephagy, and eliminated proteotoxicity in cadmium-exposed RTCs. Pharmacological inhibition of Akt using MK2206 or Akti-1/2 also mitigated cadmium-induced cell death, and this protection was diminished by the depletion of TFEB/TFE3. Taken together, our findings constitute the first demonstration, to our knowledge, that dysregulated Akt could exert its inhibitory effects on cell survival through proteotoxicity in cadmium-exposed RTCs.

We observed that cadmium exposure induced accumulation of ubiquitinated proteins and aggresome formation in HK-2 cells, RPTECs, and RPTEC/TERT1, another human proximal tubular cell line (data not shown). It has been reported that cadmium exposure does not affect proteasome activity^51,52^. Cadmium exposure induces protein misfolding and aggregation by binding to the thiol groups of cysteine residues in cells^37^. Cadmium exposure induces reactive oxygen species (ROS) in many types of cells, and an excessive ROS causes oxidative damage to proteins. Therefore, taking these result together with our findings, we speculated that many of the ubiquitinated proteins which accumulate in aggresome might be misfolded or damaged proteins caused by the direct binding to cadmium or ROS-mediated oxidation in cadmium-exposed RTCs. However, it is also possible that proteins originally required for cellular homeostasis are erroneously ubiquitinated and transported to aggresome due to the disruption of multiple signaling network in cadmium-exposed RTCs. Therefore, further examination will be needed to confirm what proteins are ubiquitinated and accumulated in aggresome in cadmium-exposed RTCs.

Phosphorylation/dephosphorylation events play an important role in TFEB/TFE3 regulation. We observed that Akt facilitated the phosphorylation of TFEB-S211 and TFEB-S122, and delayed TFEB nuclear translocation in cadmium-exposed RTCs. TFE3 electrophoretic mobility showed that Akt might also delay TFE3 nuclear translocation via its phosphorylation in cadmium-exposed RTCs. Akt directly phosphorylates TFEB-S467 and other kinases also directly phosphorylate TFEB/TFE3 at multiple residues to activate or inactivate TFEB/TFE3^28–30^. Thus, we need to monitor the phosphorylation status of TFEB/TFE3 other than TFEB-S211 and TFEB-S122, and investigate the significance of other kinases as well as Akt and mTORC1 to elucidate the precise regulation of TFEB/TFE3 in cadmium-exposed RTCs. We also showed that cadmium exposure facilitates TFEB/TFE3 dephosphorylation without inhibiting Akt and mTORC1 activities, and that calcineurin might dephosphorylate TFEB-S122 in cadmium-exposed RTCs. Since cadmium exposure elevates intracellular Ca^2+^ levels in many types of cells and calcium ion (Ca^2+^) activates calcineurin via a calcium binding protein calmodulin, we speculate that cadmium exposure might activate calcineurin by the increase of intracellular Ca^2+^ in RTCs. Because calcineurin dephosphorylates a transcription factor nuclear factor of activated T cells (NFATC1) to facilitate its nuclear translocation^53^, we monitored the effects of cadmium exposure on nuclear transfer and electrophoretic mobility of NFATC1 in HK-2 cells to confirm this theory. Cadmium exposure increased nuclear NFATC1 levels but hardly increased electrophoretic mobility (data not shown). In contrast, both were markedly increased in HK-2 cells treated with the calcineurin activator ionomycin (data not shown). Thus, although cadmium exposure has been reported to activate calcineurin in bone mesenchymal stem cells^29^, further studies are needed to determine whether calcineurin could be activated in cadmium-exposed RTCs. Additionally, FK506 treatment did not completely block cadmium-induced dephosphorylation of TFEB and increase of TFE3 electrophoretic mobility in both HK-2 cells and RPTECs, indicating that other phosphatases or molecular mechanisms might be involved in this regulatory process. Interestingly, Nakamura et al.^34^ showed that Ca^2+^ efflux from lysosome facilitates dephosphorylation and nuclear translocation of TFEB independently of calcineurin in cells treated with a lysosomotropic compound l-leucyl-l-leucin methyl ester. In addition, hydrogen peroxide (H_2_O_2_) treatment oxidizes TFEB/TFE3/MITF at cysteine residues and blocks the association with Rag GTPase, resulting in their nuclear translocation and activation^54^. Thus, we need further examination to analyze whether these machineries could contribute to the activation of TFEB/TFE3 in cadmium-exposed RTCs. Furthermore, the possibility that PP2 contributes to the dephosphorylation of TFEB/TFE3 in cadmium-exposed RTCs cannot be completely ruled out based on the present study using okadaic acid alone.

The present study demonstrated that cadmium-induced aberrant activation of Akt promotes RTCs death by diminishing aggrephagy. Recent evidences suggest that cadmium exposure causes lysosomal dysfunction^55–57^. Consistently, although TFEB/TFE3 nuclear translocation was facilitated in cadmium-exposed cells, cadmium exposure decreased the levels of Cathepsin B and D (data not shown). Thus, lysosomal dysfunction and aggrephagy might occur simultaneously in cadmium-exposed RTCs, and suppression of Akt activity might ameliorate lysosome dysfunction and facilitate aggrephagy. In addition, much evidence indicated that autophagy has promotive and inhibitory roles for cell survival. Lee et al.^58^ reported that the state of lysosome determines whether autophagy promotes or inhibits cell survival under cadmium exposure. Our findings highlight the protective role of autophagy against the activated Akt-dependent cadmium toxicity. While in vivo studies will be necessary to further validate the reliability of our new knowledge, these results will contribute to the understanding of cadmium toxicity and to the development of therapeutic approaches for cadmium-induced renal injury. Furthermore, since TFEB reportedly has a protective role against renal fibrosis, cisplatin nephropathy, renal ischemia/reperfusion injury, or diabetic nephropathy in mouse models^34,59–61^, it might be possible that the role and mechanism of the Akt/TFEB/TFE3 axis in cadmium toxicity that we have proposed are conserved in other renal diseases.

## Methods

### Chemicals

CdCl_2_ and chloroquine diphosphate were obtained from Wako Pure Chemical Industries, Ltd. (Osaka, Japan). Akti-1/2 and MK2206 were obtained from MedChemExpress (Monmouth Junction, NJ, USA). Nocodazole, Torin, rapamycin, FK506, and okadaic acid were obtained from Cayman Chemical Company (Ann Arbor, MI, USA). Antibodies against LC3B, LC3B (D11) XP®, LC3B (E5Q2K), phospho-Akt (Thr308) (C31E5E), phospho-Akt (Ser473) (DQE) XP®, Akt (pan) (C67E7), phospho-p70 S6 kinase (Thr-389) (108D2), phospho-TFEB (Ser211) (EQS8N), phospho-TFEB (Ser122), TFEB, Cathepsin B (D1C7Y) XP®, Cathepsin D (E179), phospho-FoxO1 (Tr24)/FoxO3a (Thr32), FOXO3a (75D8), Vimentin (D21H3) XP®, and HDAC6 (D2E5) were obtained from Cell Signaling Technology, Inc. (Beverly, MA, USA). Multi-Ubiquitin antibody was obtained from MBL (Tokyo, Japan). TFE3 antibody was obtained from Sigma, Merck KGaA (Darmstadt, Germany). Pericentrin antibody was obtained from abcam plc (Cambridge, England). Actin (I-19), PCM1 (G-6), and c-Myc (9E10) antibodies were obtained from Santa Cruz Biotechnology, Inc. (Santa Cruz, CA, USA). GAPDH (GT239) and MITF antibodies were obtained from Genetex Inc. (Irvine, CA, USA). The siRNAs targeted against the human *TFEB* (siRNA-1: HSS111868 Stealth siRNA, siRNA-2: HSS111870 Stealth siRNA), *TFE3* (siRNA-1: s14031 Silencer™ Select siRNA, siRNA-2: s14031 Silencer™ Select siRNA), *MITF* (siRNA: s8792 Silencer™ Select siRNA), Silencer™ select Negative Control No.1 siRNA, and Stealth RNAi™ siRNA Negative Control Med GC were purchased from Thermo Fisher Scientific. pcDNA3.1-TFEB-WT-MYC (Addgene plasmid 99955) was a gift from James Brugarolas. pEGFP-N1-TFE3 (Addgene plasmid 38120) and pEGFP-N1-MITF-D (Addgene plasmid 38133) were gifts from Shawn Ferguson.

### Cell culture and treatments

HK-2 cells were obtained from the American Type Culture Collection (Manassas, VA, USA) and grown in Dulbecco’s modified Eagle’s medium/Nutrient Mixture F-12 supplemented with 10% heat-inactivated fetal bovine serum, 100 U/ml penicillin, and 100 µg/ml streptomycin (GIBCO, Invitrogen Corp., Carlsbad, CA, USA) in a humidified atmosphere of 5% CO_2_ and 95% air at 37 °C. Exponentially growing HK-2 cells were seeded at 1.9–4 × 10^5^ cells/well in six-well culture plates and cultured for 1 day before each experiment. CdCl_2_ was dissolved in water and sterilized by filtration. Cells were incubated in serum-free medium containing the appropriate concentration of CdCl_2_ for 1–36 h at 37 °C. Rapamycin, Torin, MK-2206, Akti-1/2, nocodazole, FK506, and okadaic acid were dissolved in DMSO. After pretreatment with each inhibitor for 0.5 or 1 h, HK-2 cells were treated with 25 µM CdCl_2_ for the indicated time. Primary human renal proximal tubule epithelial cells (RPTECs) were obtained from Lonza (Basel, Switzerland), and grown in REGM™ Renal Epithelial Cell Growth Medium supplemented with 100 U/ml penicillin and 100 µg/ml streptomycin. RPTECs were treated with inhibitors or CdCl_2_ in the same methods as in HK-2 cells.

### Preparation of whole cell lysates

After incubation, cells were washed with phosphate-buffered saline and lysed with sodium dodecyl sulfate–polyacrylamide gel Laemmli sample buffer. Cell lysates were collected, sonicated, and boiled for 5 min. Protein concentrations were determined using the RC DC Protein Assay (Bio-Rad Laboratories, Inc., Hercules, CA, USA).

### Western blotting

Equal amounts of protein (20 μg) were subjected to sodium dodecyl sulfate-4–20%, 7%, 10%, or 12% polyacrylamide gel electrophoresis and transferred onto a nitrocellulose membrane (Hybond-ECL, Amersham Pharmacia Biotech, Buckinghamshire, England). The membrane was blocked with 5% non-fat milk in Tris-buffered saline containing 0.1% Tween 20 for 1 h at room temperature. The membrane was then incubated overnight at 4 °C with the primary antibody, and protein was detected with a Phototope-HRP Western blot detection kit (Cell Signaling Technology, Inc.). The bands on the developed film were quantified using ImageJ 1.42 (National Institutes of Health, Bethesda, MD). The density of each band was normalized to the density of actin or GAPDH. The band intensities of Cathepsin D or Cathepsin B in Figures represented the immunoblots of Cathepsin D at 43 kDa and 46 kDa or Cathepsin B at 44 kDa, respectively. The displayed blots were cropped, and the full-length blots are shown in Supplementary Fig. S1. Because the full-length membranes were cut prior to hybridization with the antibodies, the full-length blots of the cut membranes are shown in Supplementary Fig. S1.

### Gene knockdown of TFEB, TFE3, and MITF by siRNA

Transfection of siRNAs against human TFEB, TFE3, and MITF, and non-target siRNA into HK-2 cells was carried out using Lipofectamine RNAiMAX (Invitrogen Corp.) according to the manufacturer’s instructions with some adjustments. The siRNAs were dissolved in nuclease-free water and diluted to 0.1 or 0.2 µM with 250 µl Opti-MEM (Invitrogen Corp.). Five microliters of Lipofectamine RNAiMAX was also diluted 50-fold with Opti-MEM. Equal volumes of these two solutions were mixed (500 µl total) and immediately added to 2 ml culture medium at the time of cell plating. After incubation for 24 h, cells were washed with medium and used for experiments.

### Plasmid transfection into HK-2

HK-2 cells were plated at 3–4 × 10^5^ cells/well in six-well culture plates, growing overnight, and transfected with each plasmid in the presence of 2.0 µg of the indicated DNA combinations with Lipofectamine3000 (Thermo Fisher Scientific) according to the manufacturer’s instructions.

### Phosphatase assay

After incubation, cells were washed with phosphate-buffered saline and lysed in RIPA buffer [50 mM Tris–HCl pH 7.4, 0.15 M NaCl, 1% Sodium Deoxycholate, 1% NP-40, 1 mM EDTA, 1 mM dithiothreitol, and protease inhibitor cocktail (Nacalai Tesque, Inc, Kyoto Japan)], followed by centrifugation at 15,000*g* for 12 min. The supernatant was collected and used for phosphatase assay. Phosphatase assay was performed by Lambda Protein Phosphatase (New England BioLabs, MA, USA) following the manufacturer’s instructions.

### Trypan blue exclusion assay

Culture medium was aspirated and reserved. After trypsinization, cells were suspended in Dulbecco’s modified Eagle’s medium/Nutrient Mixture F-12 medium or phosphate-buffered saline, and the culture medium was returned. The mixture was centrifuged to pellet the cells. Cellular suspension and 0.4% trypan blue in Hank’s Balanced Salt Solution were mixed, and the number of viable cells was counted using a TC10TM Automated Cell Counter (Bio-Rad laboratories, Inc.).

### Fluorescence microscopy and image analysis

For immunofluorescence microscopy, cells grown on coverslips were treated with drugs, and then fixed in 4% paraformaldehyde for 15 min at room temperature. Cells were then permeabilized in 1% bovine serum albumin and 0.3% Triton X100 for 1 h, and incubated with primary and secondary antibodies. Primary antibodies were rabbit anti-Pericentrin at 1:300, mouse anti-PCM1 at 1:100, rabbit anti-HDAC6 at 1:200, rabbit anti-LC3B at 1:200, mouse anti-LC3B at 1:400, rabbit anti-LAMP1 at 1:200, mouse anti-Multi-Ubiquitin at 1:200, mouse anti-Myc at 1:200, and rabbit anti-Vimentin at 1:200. Secondary antibodies were Alexa Fluor 488- and 647-conjugated donkey anti-mouse-IgG or anti-rabbit-IgG antibodies, respectively (Themo Fisher Scientific). Stained cells were photographed using a Nikon Eclipse Ti2E microscope (Nikon, Tokyo, Japan) and cell image analyzer (CellInsight, Thermo Fisher Scientific) equipped with a 20 × objective lens. The number of puncta was visually measured using software platform, NIS elements viewer (Nikon, Tokyo, Japan). For each experiment, the microscope settings were optimized for the brightest.

### Statistical analysis

Results are expressed as the mean ± SD. Statistical significance was determined by Student’s *t*-test or Welch’s *t*-test. A value of *P* < 0.05 was considered to be statistically significant.

### Supplementary Information

Supplementary Figures.

## Data Availability

The datasets used and/or analysed during the current study available from the corresponding author, [K.F.], on reasonable request.

## Abbreviations

ALK: Activin receptor-like kinase
LC3B: Microtubule-associated protein 1 light chain 3 beta
CdCl_2_: Cadmium chloride
CLEAR: Coordinated lysosomal expression and regulation
DMSO: Dimethyl sulfoxide
FOXO: Forkhead box O
HDAC: Histone deacetylase
LAMP: Lysosome associated membrane protein
MITF: Mirophthalmia-associated transcription factor
MiT/TFEs: Microphthalmia family of transcription factors
MTOC: Microtubule organizing center
mTORC: Mechanistic target of rapamycin complex
NFATC: Nuclear factor of activated T cells
PP2: Protein phosphatase 2
RPTECs: Primary human renal proximal tubule epithelial cells
RTCs: Renal tubular cells
ROS: Reactive oxygen species
S6K: S6 kinase
TFE3: Transcription factor E3
TFEB: Transcription factor EB
TGF: Transforming growth factor
UPS: Ubiquitin–proteasome system
