# Supplementary material for: Blockage of Akt activation suppresses cadmium-induced renal tubular cellular damages through aggrephagy in HK-2 cells
Source: Sci Rep. 2024 Jun 24;14:14552. doi: 10.1038/s41598-024-64579-3 (PMC11196260; doi:10.1038/s41598-024-64579-3)

Blockage of Akt activation suppresses cadmium-induced renal tubular cellular damages through aggrephagy in HK-2 cells

K Fujiki[1]*, K Tanabe[2], S Suzuki[3], A Mochizuki[4], M Mochizuki-Kashio[5], T Sugaya[6], T Mizoguchi[3], M Itoh[3], A Nakamura-Ishizu[5], H Inamura[1], and M Matsuoka[1]

[1]Department of Hygiene and Public Health, Tokyo Women's Medical University, Tokyo 162-8666, Japan
[2]Institute for Comprehensive Medical Sciences, Tokyo Women's Medical University, Tokyo 162-8666, Japan
[3]Graduate School of Pharmaceutical Sciences, Chiba University, Chiba 260-8675, Japan
[4]Department of Bio-Medical Engineering, School of Engineering, Tokai University, Kanagawa 259-1143, Japan
[5]Department of Microanatomy and Development Biology, Tokyo Women's Medical University, Tokyo 162-8666, Japan
[6]Division of Nephrology and Hypertension, St. Marianna University School of Medicine, Kanagawa 216-8511, Japan

*Corresponding author

Supplemental Fig. S1: Full-length blots of Fig. 1-6

Fig. 1

----- membrane cutting line

blotting area used for data in the result section

M: marker (The positions of the molecular markers were plotted on the x-ray film with a black ink pen.)

a

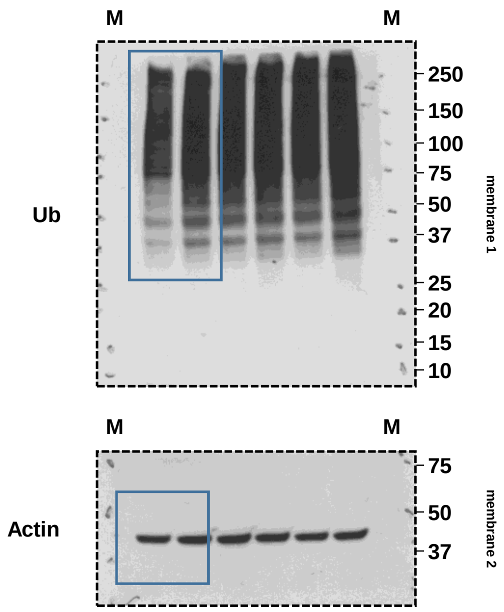

Fig. 2

----- membrane cutting line

blotting area used for data in the result section

M: marker (The positions of the molecular markers were plotted on the x-ray film with a black ink pen.)

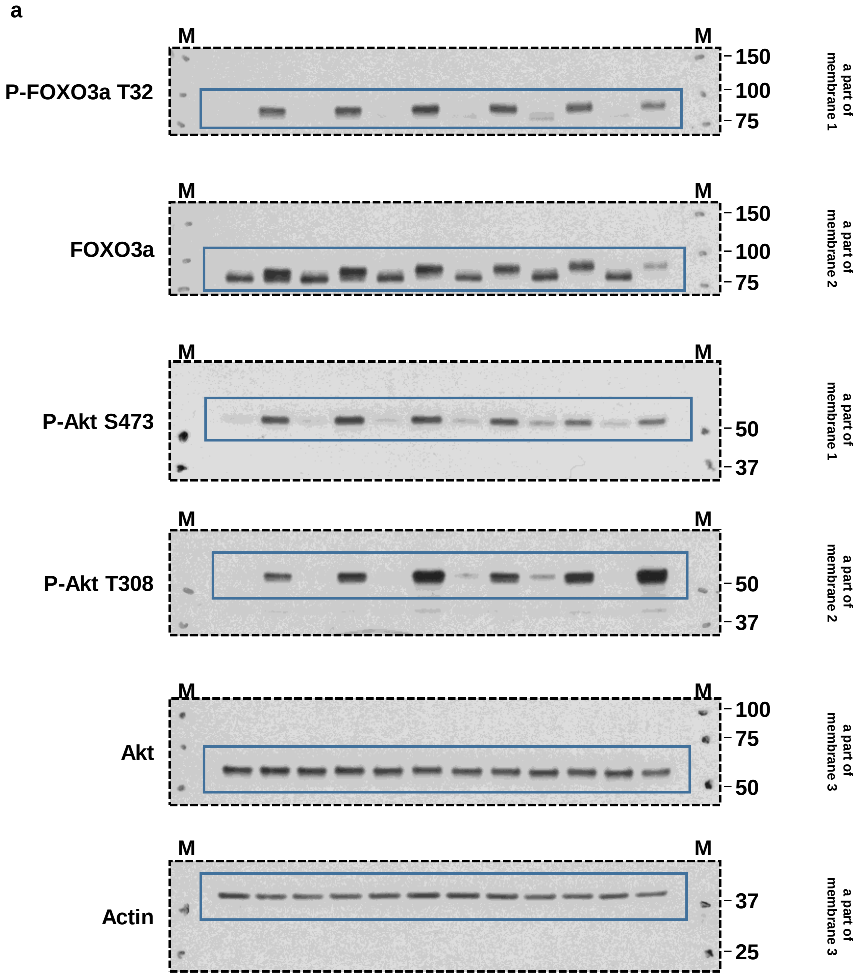

Fig. 2

----- membrane cutting line

blotting area used for data in the result section

M: marker (The positions of the molecular markers were plotted on the x-ray film with a black ink pen.)

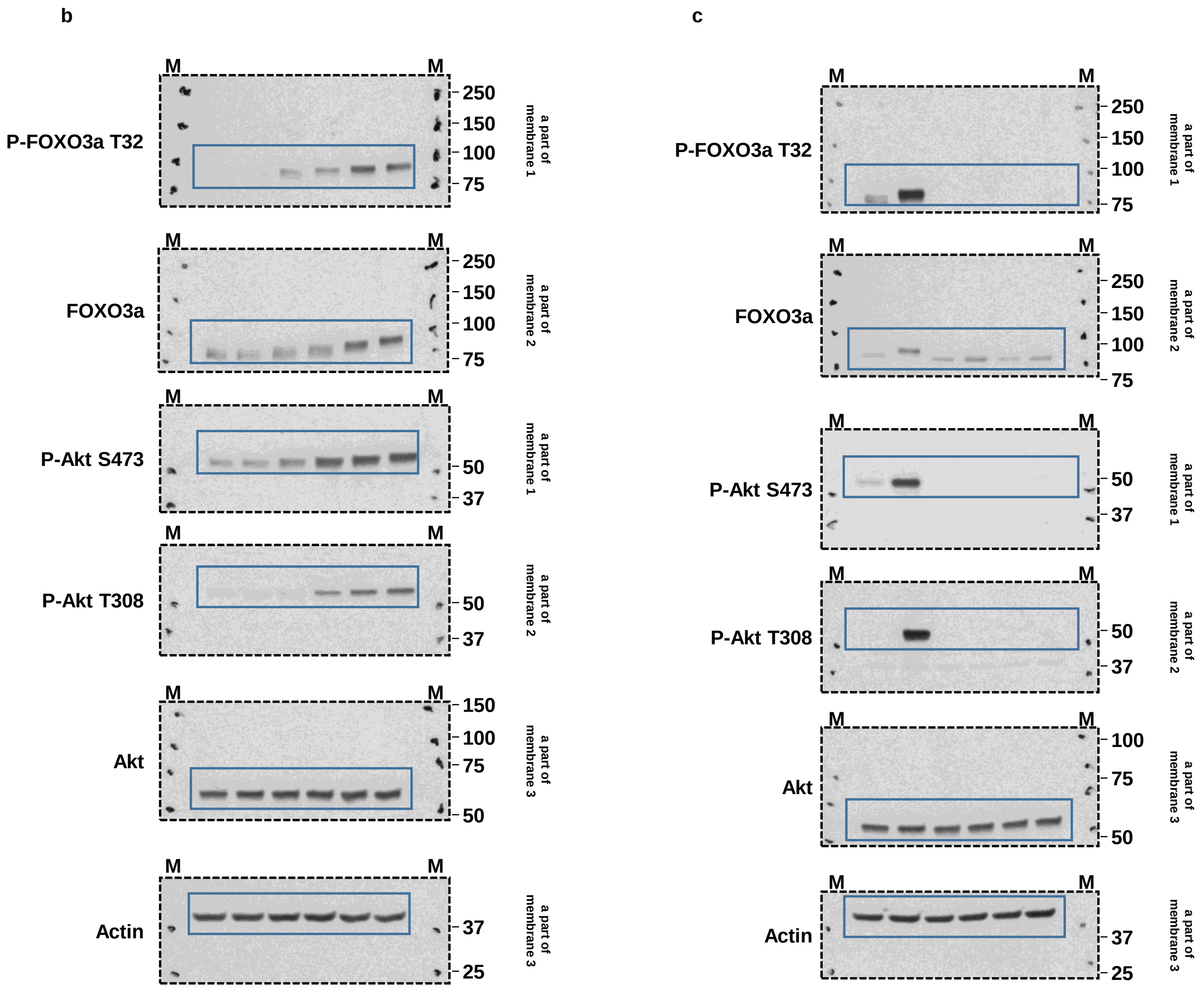

Fig. 3

----- membrane cutting line

▢ blotting area used for data in the result section

M: marker (The positions of the molecular markers were plotted on the x-ray film with a black ink pen.)

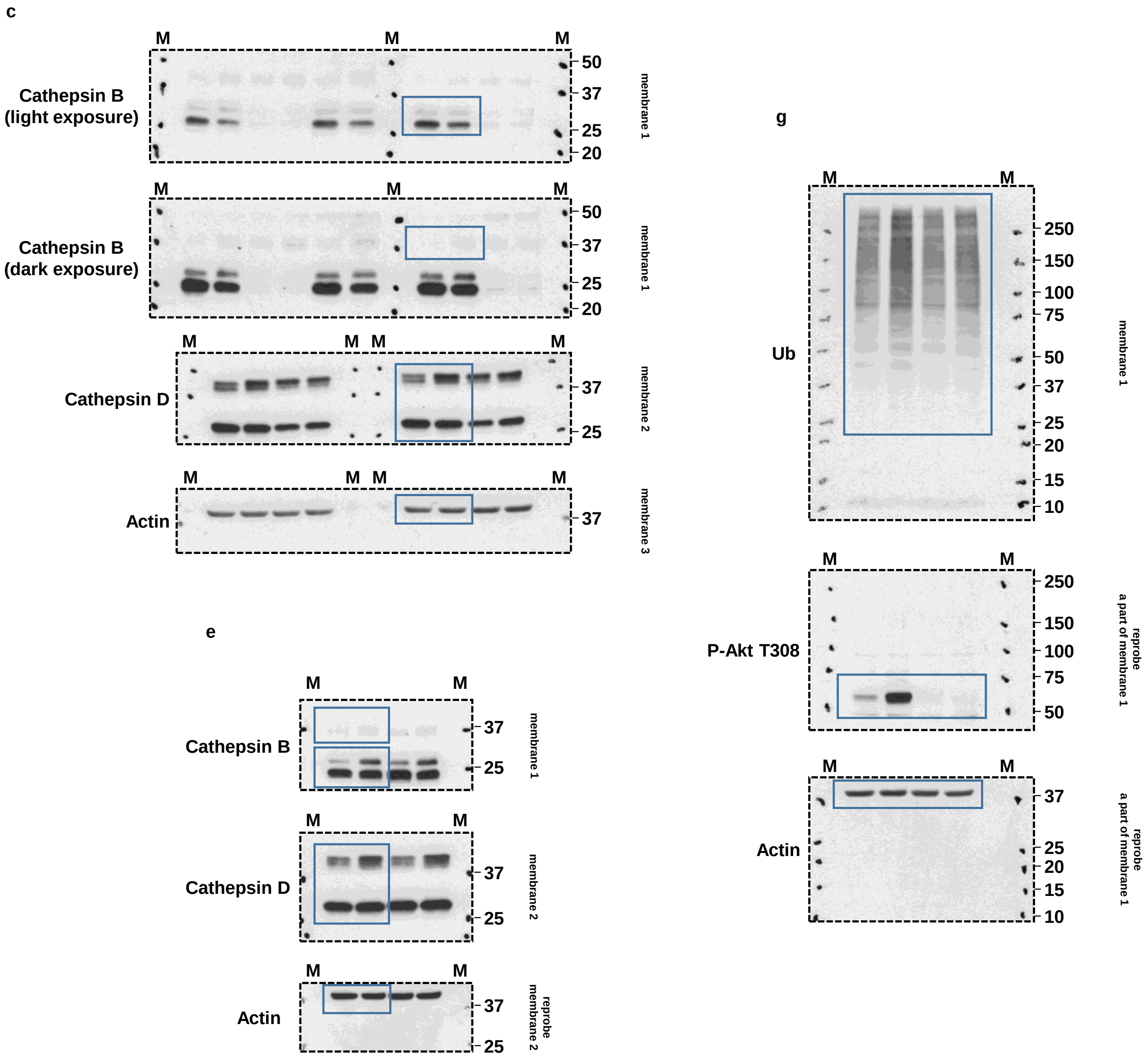

Fig. 3

----- membrane cutting line

blotting area used for data in the result section

M: marker (The positions of the molecular markers were plotted on the x-ray film with a black ink pen.)

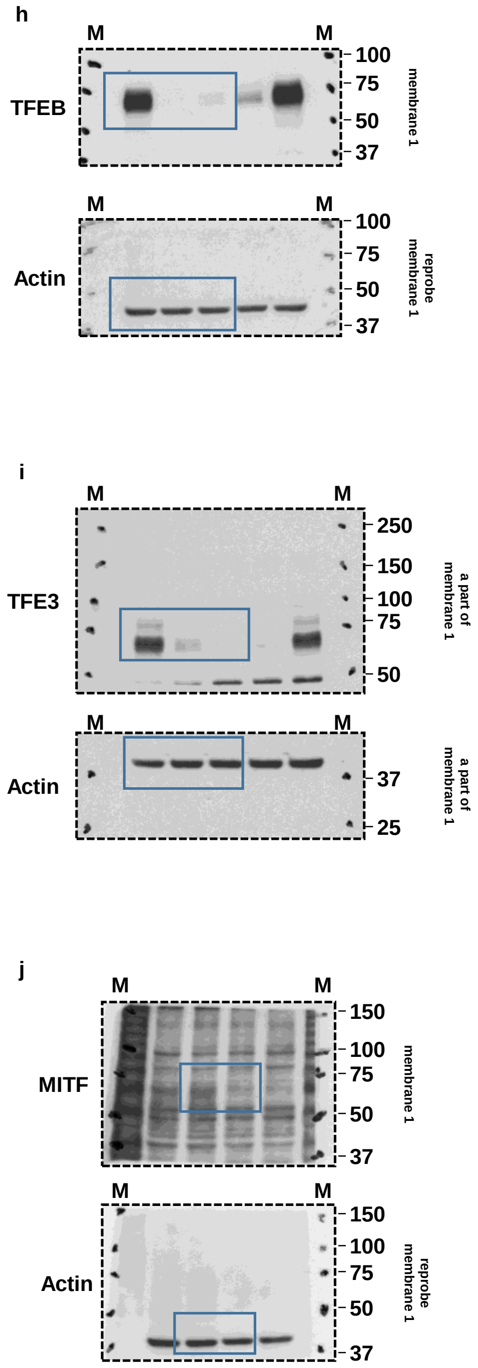

Fig. 4

----- membrane cutting line

☐ blotting area used for data in the result section

M: marker (The positions of the molecular markers were plotted on the x-ray film with a black ink pen.)

a

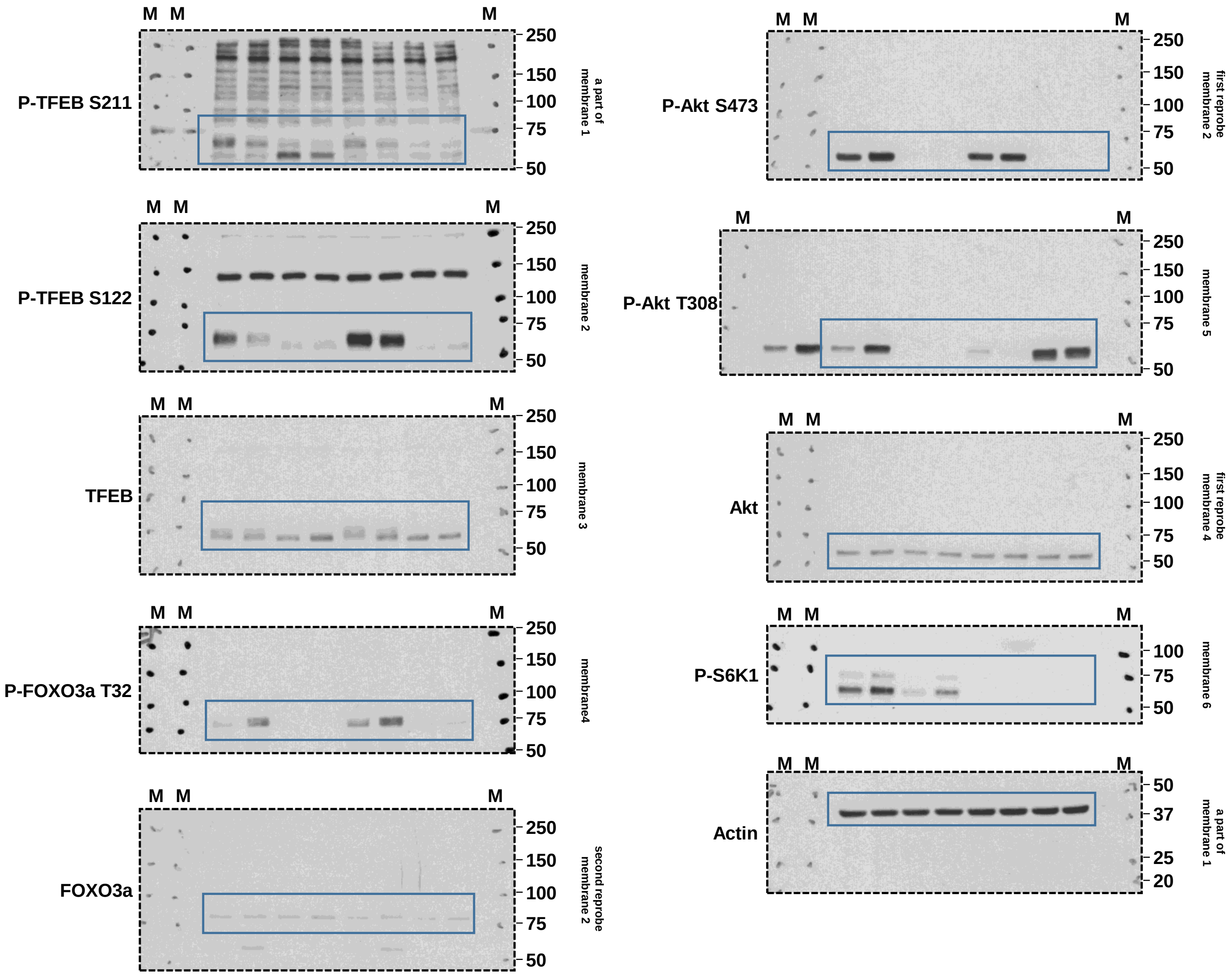

Fig. 4

----- membrane cutting line

blotting area used for data in the result section

M: marker (The positions of the molecular markers were plotted on the x-ray film with a black ink pen.)

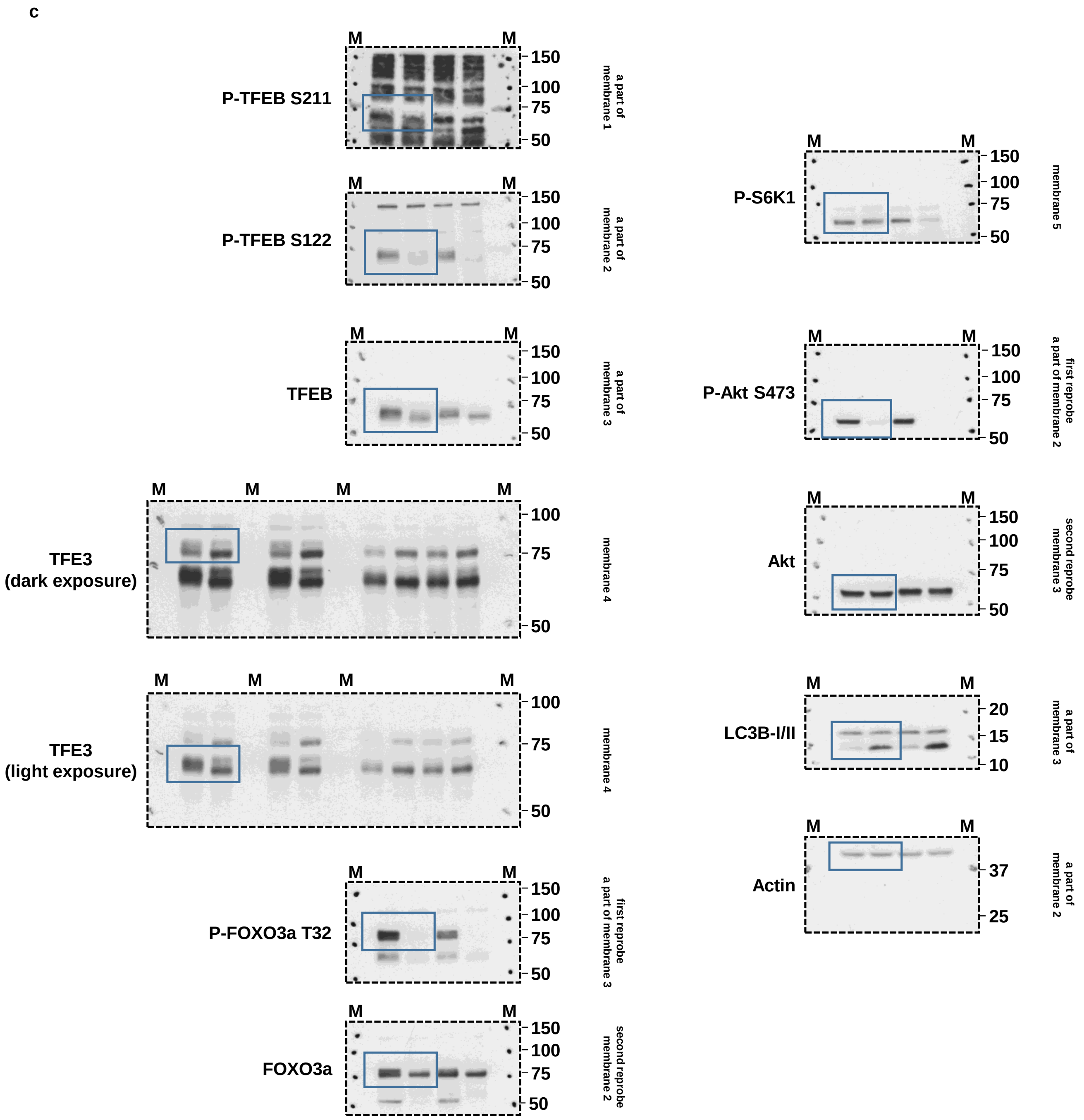

Fig. 4

----- membrane cutting line

☐ blotting area used for data in the result section

M: marker (The positions of the molecular markers were plotted on the x-ray film with a black ink pen.)

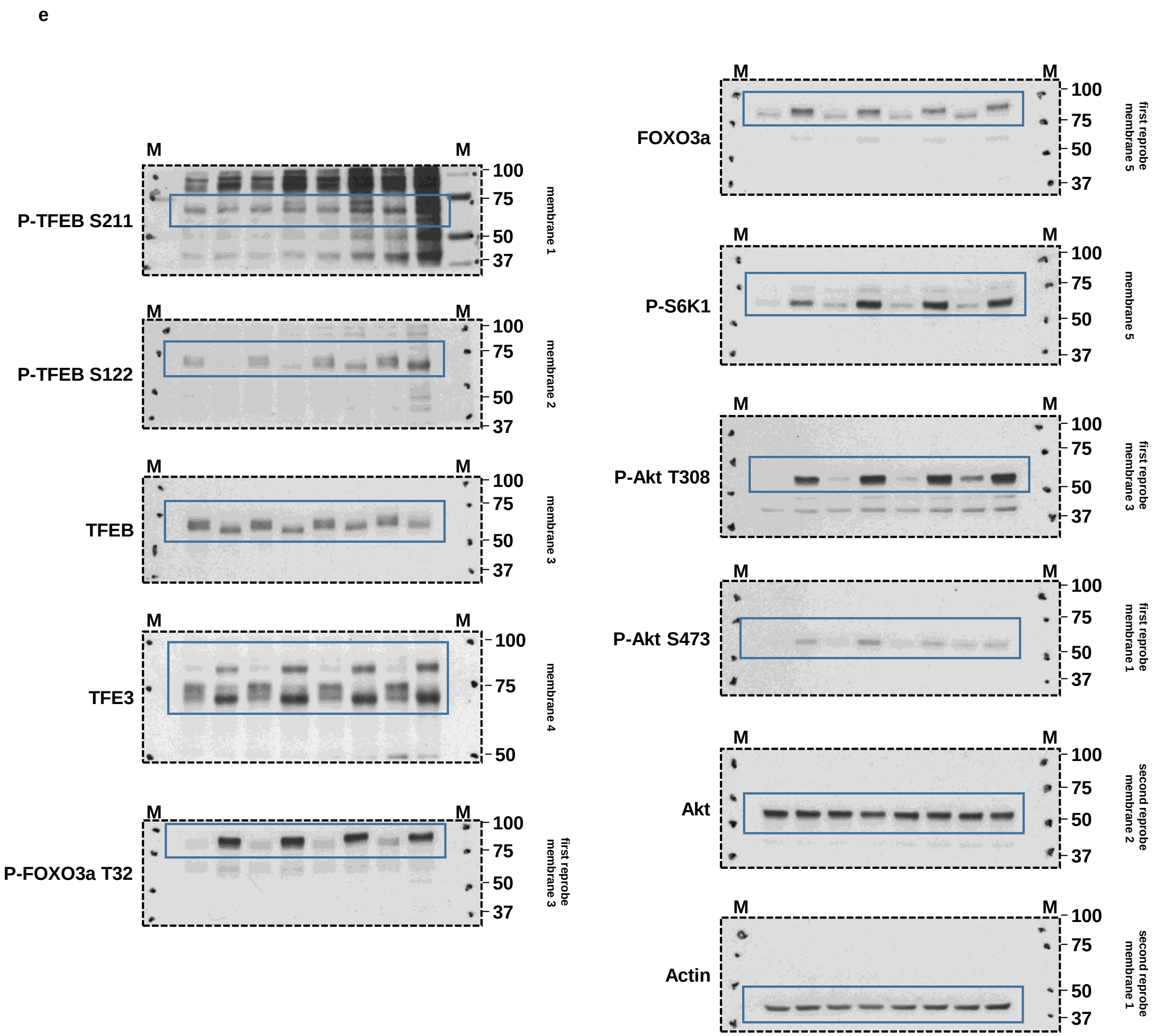

Fig. 4

----- membrane cutting line

☐ blotting area used for data in the result section

M: marker (The positions of the molecular markers were plotted on the x-ray film with a black ink pen.)

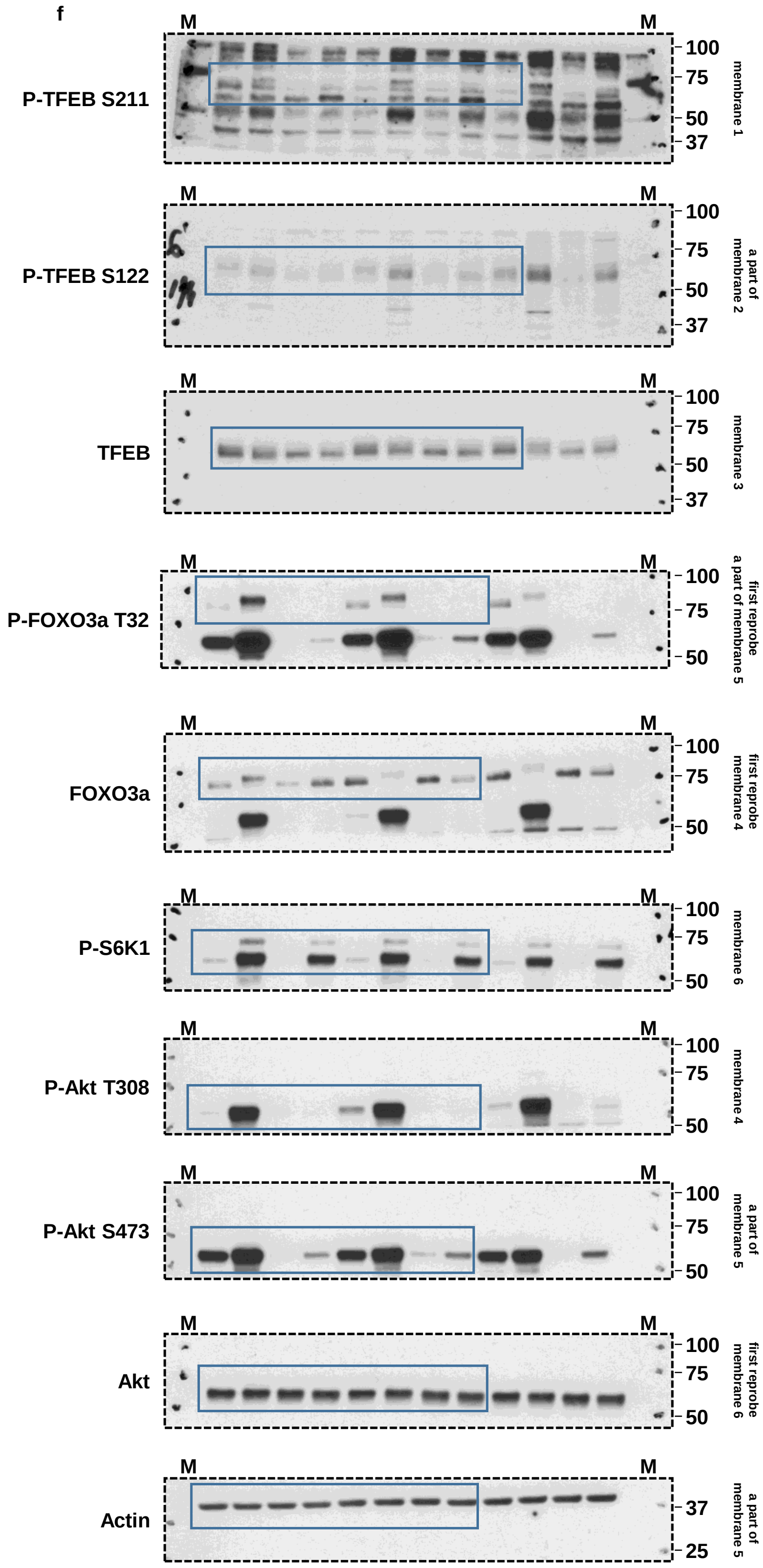

Fig. 4

----- membrane cutting line

blotting area used for data in the result section

M: marker (The positions of the molecular markers were plotted on the x-ray film with a black ink pen.)

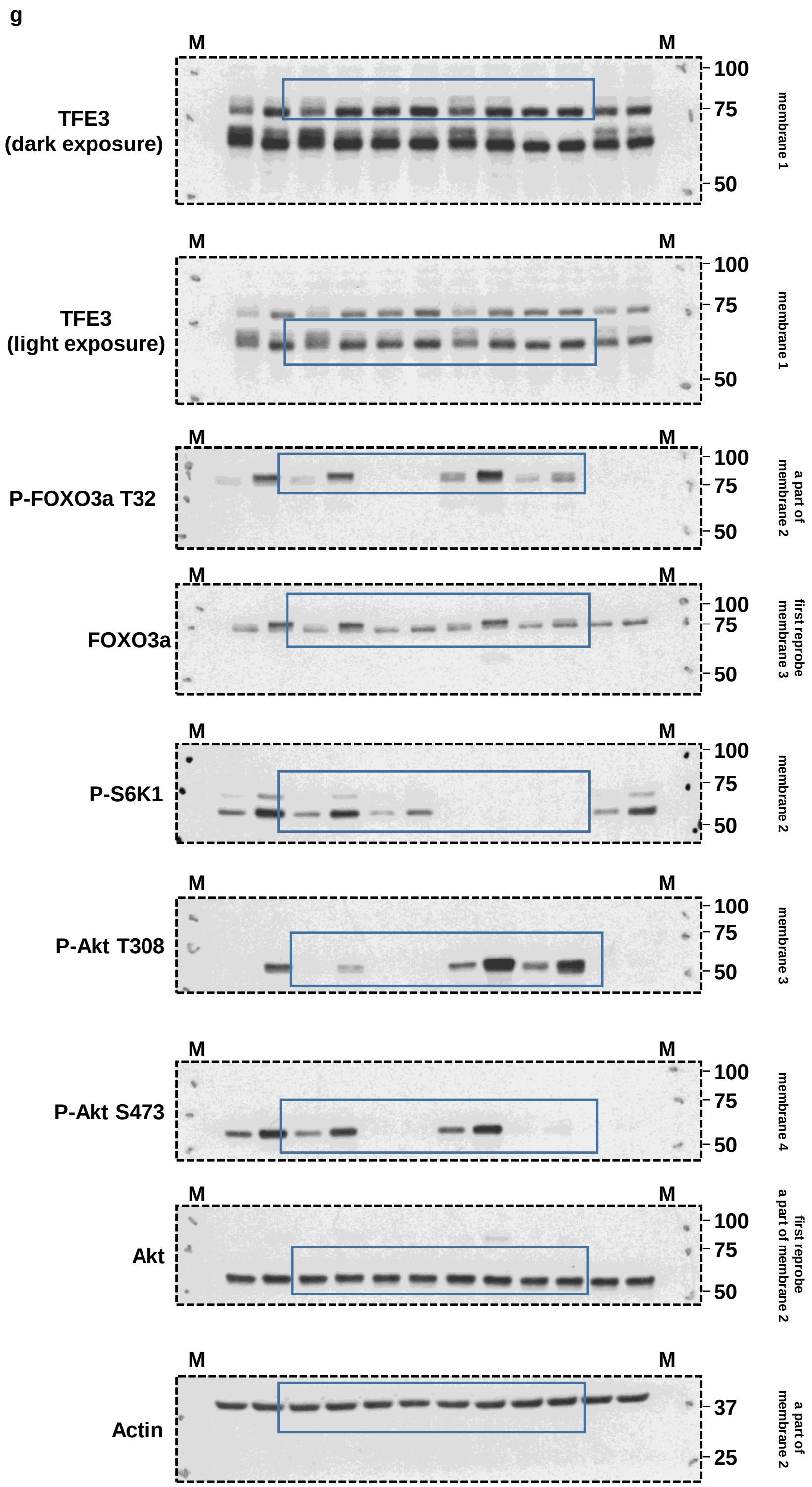

Fig. 4

----- membrane cutting line

blotting area used for data in the result section

M: marker (The positions of the molecular markers were plotted on the x-ray film with a black ink pen.)

h

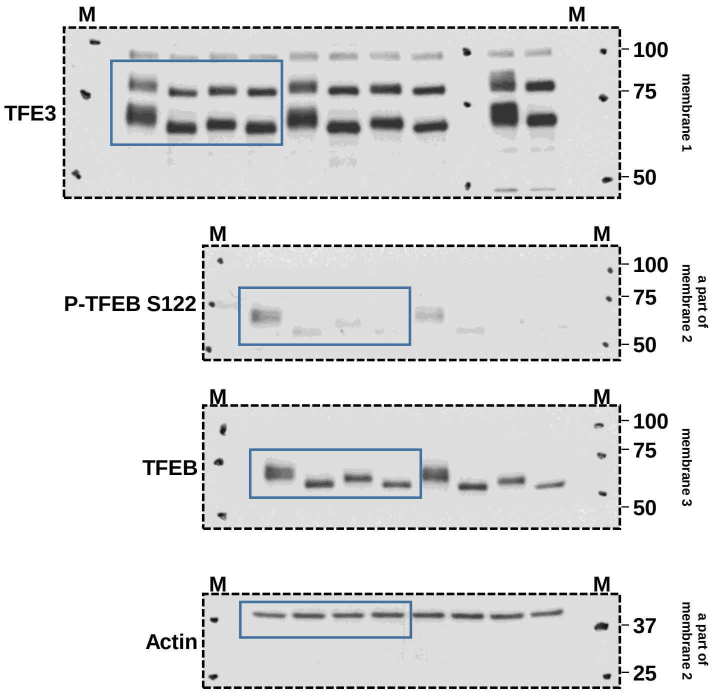

Fig. 5

----- membrane cutting line

blotting area used for data in the result section

M: marker (The positions of the molecular markers were plotted on the x-ray film with a black ink pen.)

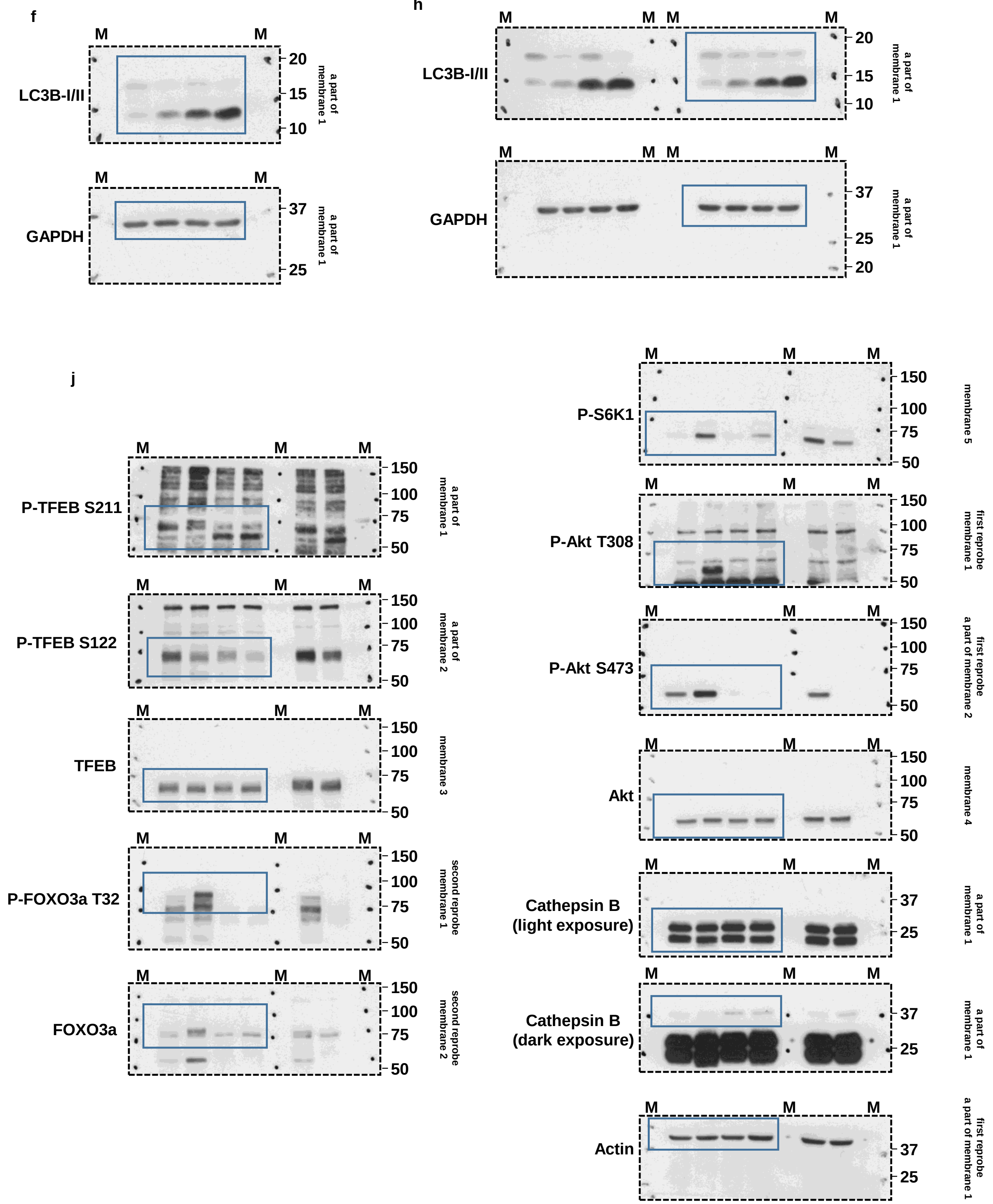

Fig. 5

----- membrane cutting line

☐ blotting area used for data in the result section

M: marker (The positions of the molecular markers were plotted on the x-ray film with a black ink pen.)

I

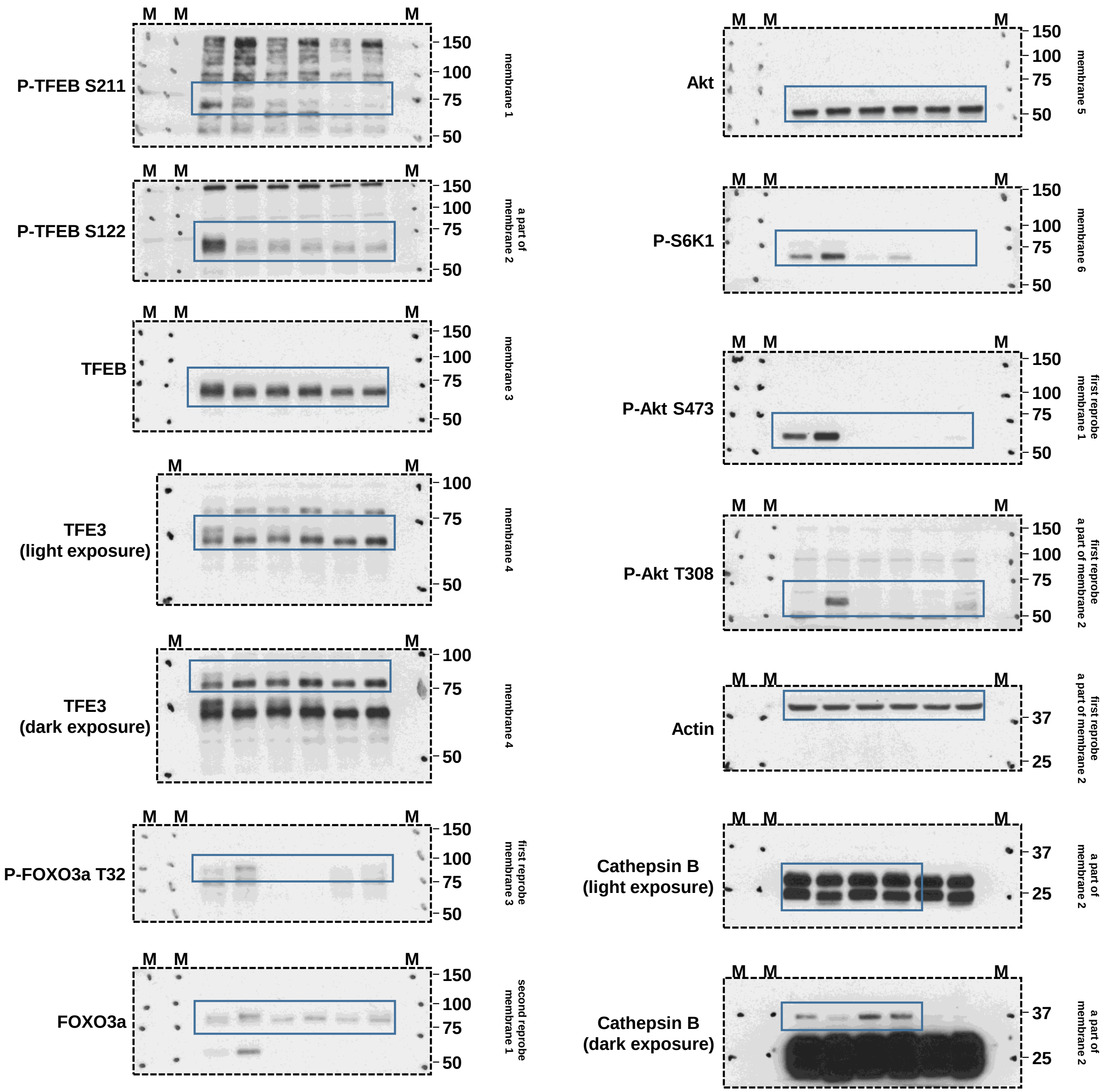

Fig. 5

----- membrane cutting line

□ blotting area used for data in the result section

M: marker (The positions of the molecular markers were plotted on the x-ray film with a black ink pen.)

n

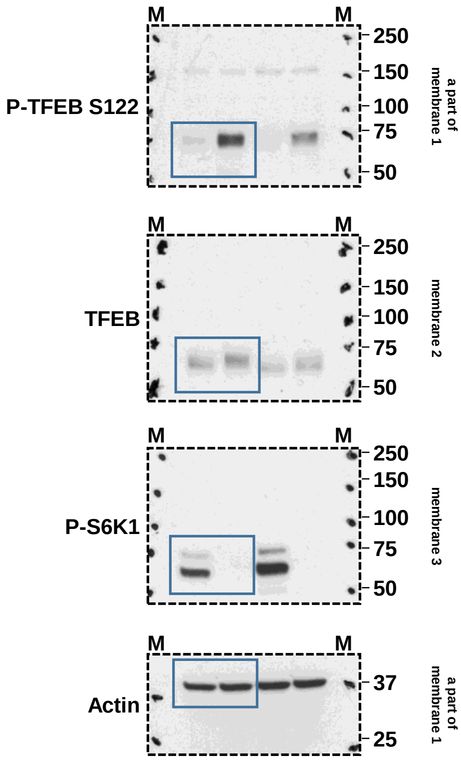

Fig. 5

----- membrane cutting line

☐ blotting area used for data in the result section

M: marker (The positions of the molecular markers were plotted on the x-ray film with a black ink pen.)

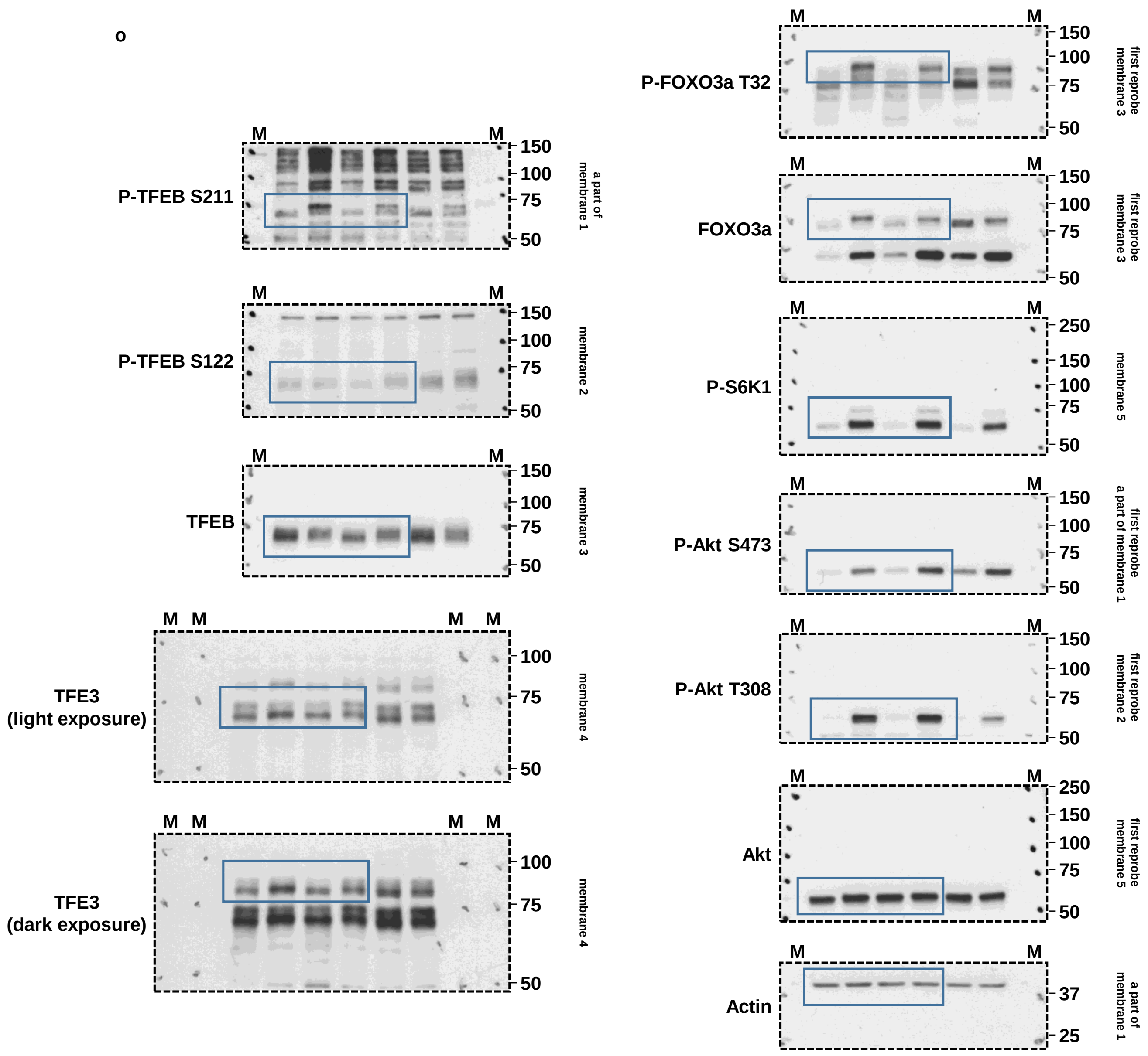

Fig. 6

----- membrane cutting line

blotting area used for data in the result section

M: marker (The positions of the molecular markers were plotted on the x-ray film with a black ink pen.)

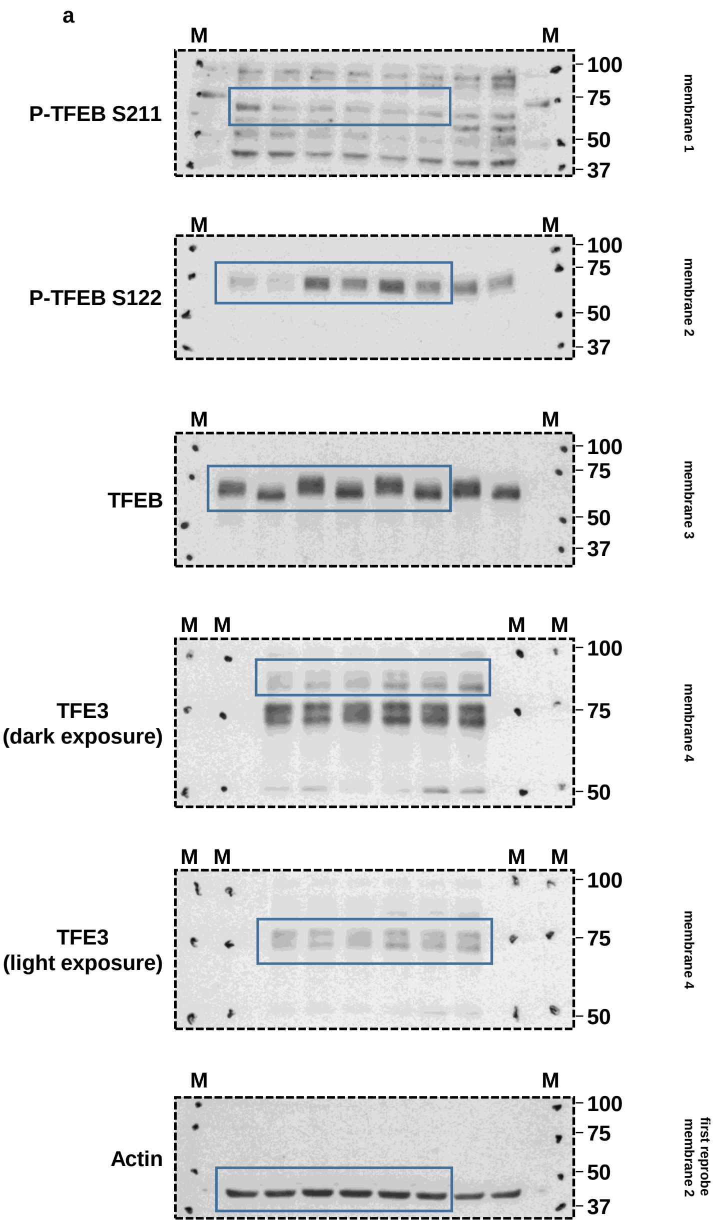

Fig. 6

----- membrane cutting line

blotting area used for data in the result section

M: marker (The positions of the molecular markers were plotted on the x-ray film with a black ink pen.)

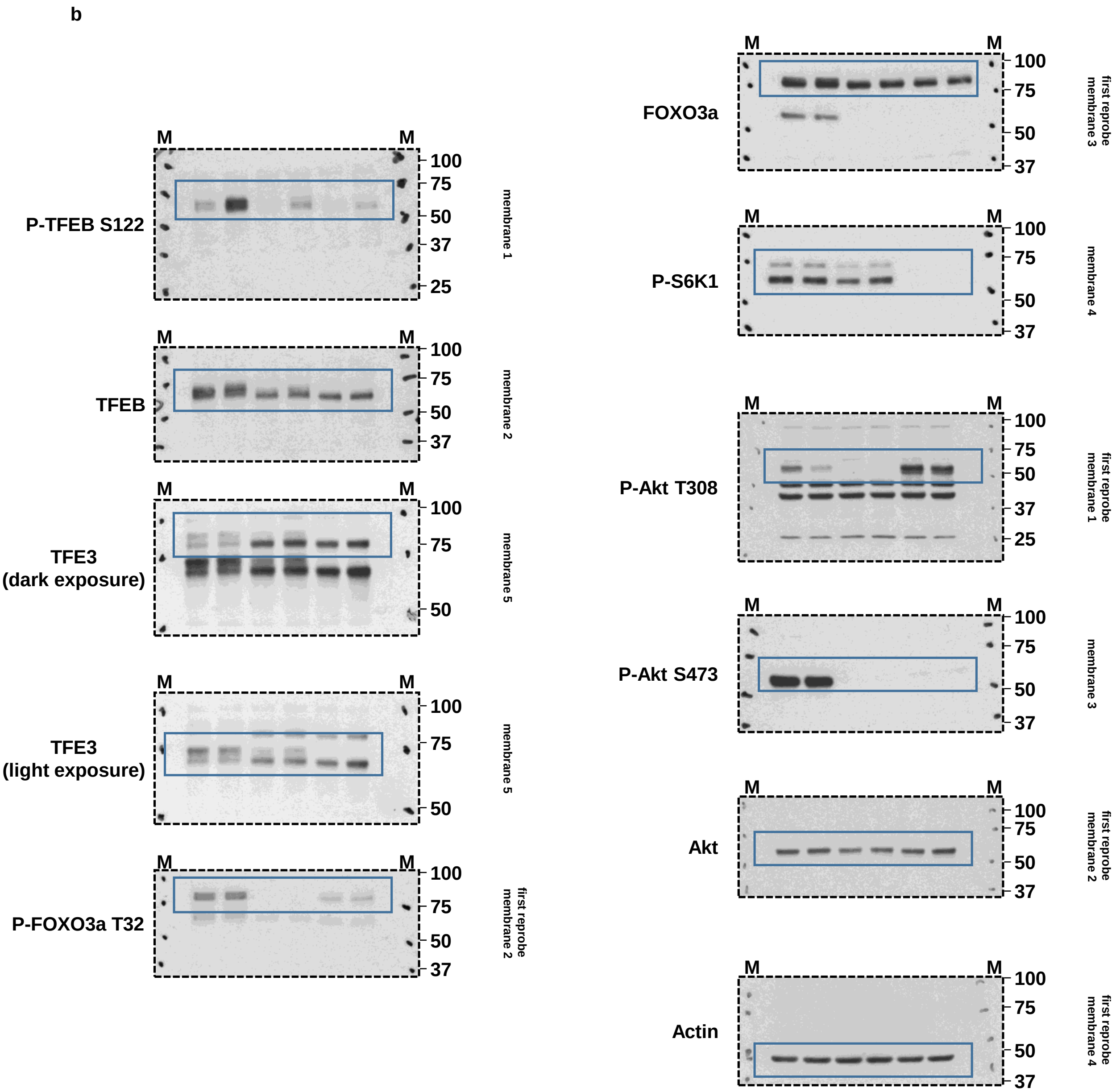

Fig. 6

----- membrane cutting line

☐ blotting area used for data in the result section

M: marker (The positions of the molecular markers were plotted on the x-ray film with a black ink pen.)

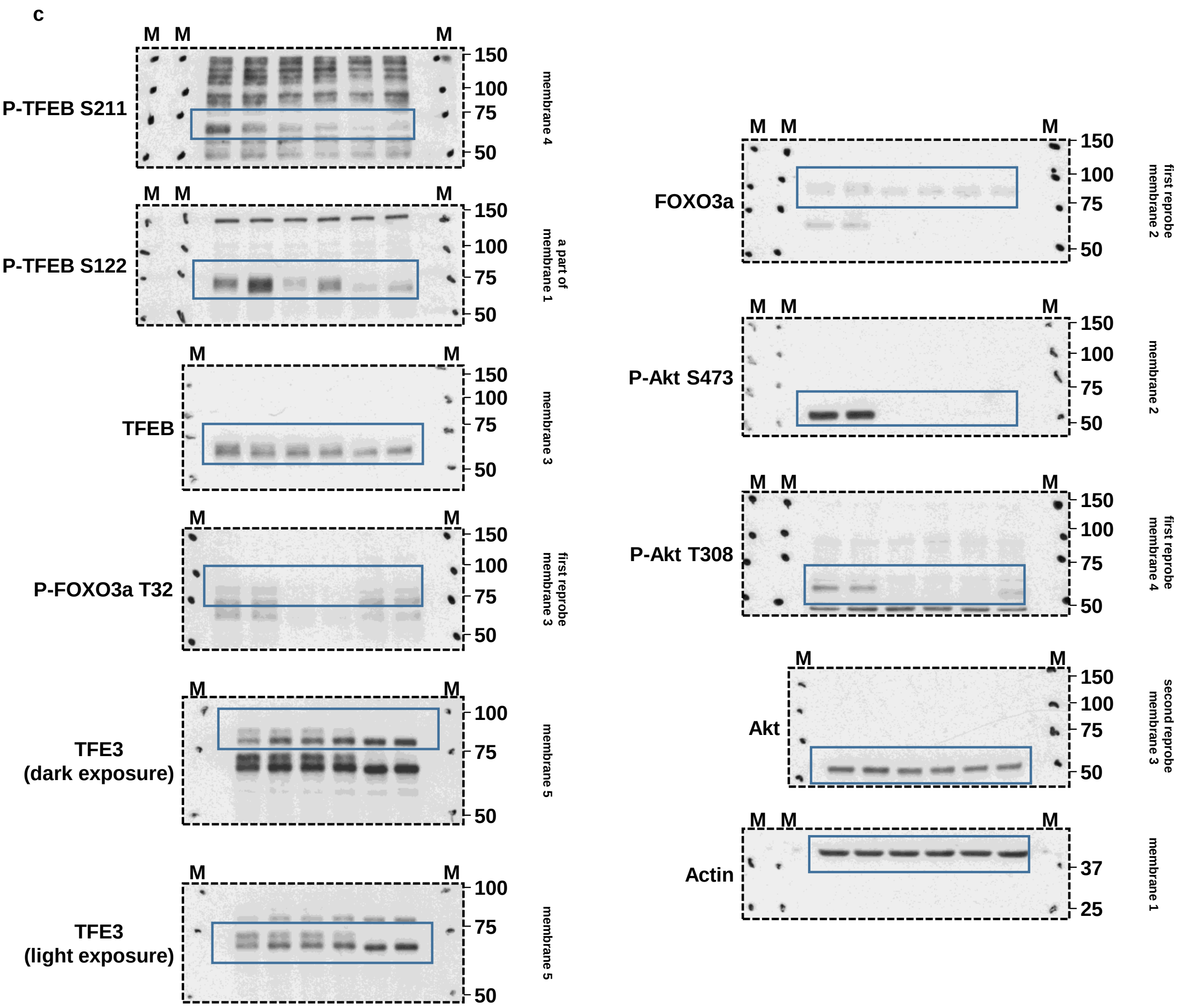

Fig. 6

----- membrane cutting line

blotting area used for data in the result section

d

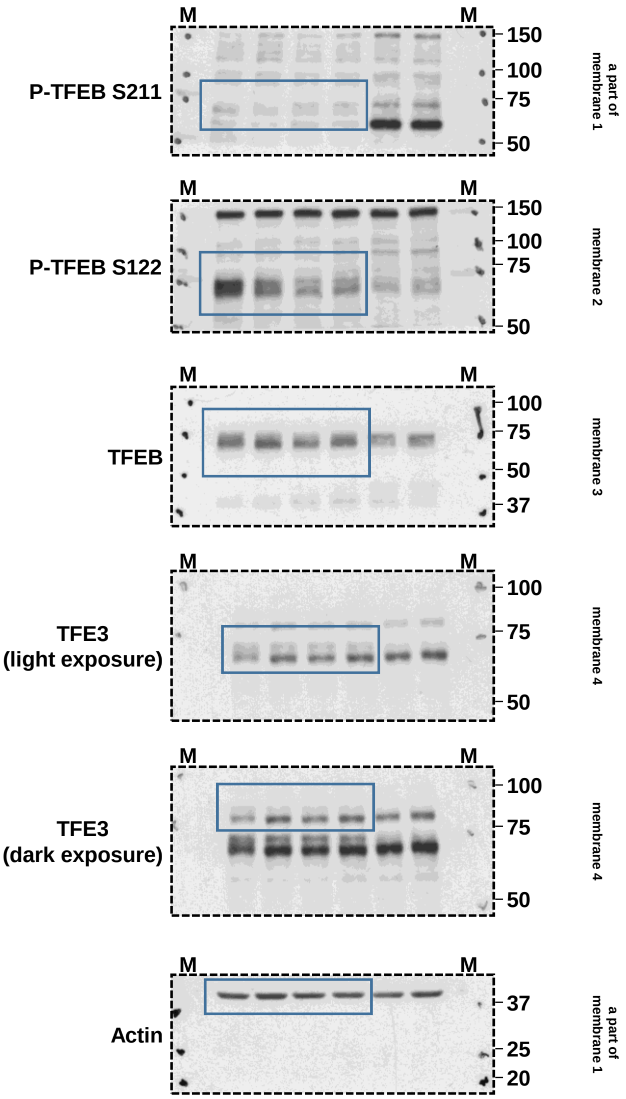

Supplement: Supplementary file 1 — Supplementary Figures. [file 41598_2024_64579_MOESM1_ESM.pdf]
